# Robustness of signal detection in cryo-electron microscopy via a bi-objective-function approach

**DOI:** 10.1186/s12859-019-2714-8

**Published:** 2019-04-03

**Authors:** Wei Li Wang, Zhou Yu, Luis R. Castillo-Menendez, Joseph Sodroski, Youdong Mao

**Affiliations:** 1 Intel® Parallel Computing Center for Structural Biology, Dana-Farber Cancer Institute, Boston, MA 02215 USA; 2 Department of Cancer Immunology and Virology, Dana-Farber Cancer Institute, Department of Microbiology, Harvard Medical School, Boston, MA 02115 USA; 3 State Key Laboratory of Artificial Microstructures and Mesoscopic Physics, School of Physics, Center for Quantitative Biology, Peking University, Beijing, 100871 China; 4 Graduate School of Arts and Sciences, Department of Cellular and Molecular Biology, Harvard University, Cambridge, MA 02138 USA; 5 Department of Immunology and Infectious Diseases, Harvard T.H. Chan School of Public Health, Boston, MA 02115 USA

**Keywords:** Automatic particle picking, Fast local correlation function, Cryo-EM, Maximum-likelihood estimate, Single-particle analysis

## Abstract

**Background:**

The detection of weak signals and selection of single particles from low-contrast micrographs of frozen hydrated biomolecules by cryo-electron microscopy (cryo-EM) represents a major practical bottleneck in cryo-EM data analysis. Template-based particle picking by an objective function using fast local correlation (FLC) allows computational extraction of a large number of candidate particles from micrographs. Another independent objective function based on maximum likelihood estimates (MLE) can be used to align the images and verify the presence of a signal in the selected particles. Despite the widespread applications of the two objective functions, an optimal combination of their utilities has not been exploited. Here we propose a bi-objective function (BOF) approach that combines both FLC and MLE and explore the potential advantages and limitations of BOF in signal detection from cryo-EM data.

**Results:**

The robustness of the BOF strategy in particle selection and verification was systematically examined with both simulated and experimental cryo-EM data. We investigated how the performance of the BOF approach is quantitatively affected by the signal-to-noise ratio (SNR) of cryo-EM data and by the choice of initialization for FLC and MLE. We quantitatively pinpointed the critical SNR (~ 0.005), at which the BOF approach starts losing its ability to select and verify particles reliably. We found that the use of a Gaussian model to initialize the MLE suppresses the adverse effects of reference dependency in the FLC function used for template-matching.

**Conclusion:**

The BOF approach, which combines two distinct objective functions, provides a sensitive way to verify particles for downstream cryo-EM structure analysis. Importantly, reference dependency of the FLC does not necessarily transfer to the MLE, enabling the robust detection of weak signals. Our insights into the numerical behavior of the BOF approach can be used to improve automation efficiency in the cryo-EM data processing pipeline for high-resolution structural determination.

**Electronic supplementary material:**

The online version of this article (10.1186/s12859-019-2714-8) contains supplementary material, which is available to authorized users.

## Background

Cryo-electron microscopy (cryo-EM) has recently emerged as a mainstream approach for high-resolution structure determination of biological macromolecules [1]. Image formation in electron microscopy is understood as the weak-phase approximation of thin, electron-penetrable objects [2]. The electron image formed after the objective lens is a convolution of the exit wave function passing through the object with the point spread function of the objective lens [2]. The phase-contrast transfer function (CTF), which is the Fourier transform of the point spread function of the objective lens, gives rise to a tradeoff between the resolution and the contrast of the image [3]. To image biomolecular structures in their native states by cryo-EM, the molecules of interest are flash-frozen in a thin layer of amorphous ice suspended over holes in a perforated carbon film. Thus, the biomolecular objects are surrounded by imaging noise from electrons scattered by the amorphous ice. Another thin carbon film over the holes may also be used as a support to enrich biomolecules for cryo-EM; in this case, the carbon film adds further noise. Moreover, additional noise may be introduced in the process of electron signal transfer into the recording medium, such as detection noise in a CCD camera and electron-counting noise in a direct electron detector. The strong background ice noise, together with weak-phase approximation in image formation, results in extremely low signal-to-noise ratios (SNR), which are often in the range of 0.005–0.05. Therefore, the determination of cryo-EM structures of biomolecules at high resolution requires that a large number of single-particle images, often on the scale of hundreds of thousands to a million, are acquired, aligned and averaged to remove background image noise in signal reconstruction.

Due to the required large number of images, the selection of single-particles from noisy cryo-EM micrographs represents a major practical bottleneck. Since manual selection can be very time-consuming and is prone to errors resulting from subjective factors, a number of automated approaches have been investigated. Computerized procedures for signal detection in single-particle cryo-EM involve two steps: particle picking and particle verification [4–6]. A number of algorithms have been developed to automate template-matching procedures for particle picking. However, these procedures require subsequent manual selection of particles, in some cases with the help of data clustering to expedite the rejection of false positives [7–22]. A popular implementation of these template-matching methods is based on the cross-correlation function, in which the fast local correlation (FLC) is calculated between a template image and an equally sized local area of the cryo-EM micrograph [8, 12, 13]. A disadvantage of the FLC function lies in its sensitivity to noise, which can create false correlation peaks that do not result from real signals. Furthermore, the outcome of cross-correlation algorithms may be influenced by the alignment of noise to the template used as a reference, known as “reference bias” or “reference dependency” [23].

Maximum likelihood estimation (MLE), which exhibits reduced susceptibility to reference bias compared to the cross-correlation algorithm [24, 25], has been used to evaluate the homogeneity of the picked particles by multi-reference image alignment [26, 27]. In principle, the use of two mathematically distinct objective functions in signal recognition can serve as a test of the robustness of the image analysis and a verification of the detected signals, since reference dependency is not expected to be reproduced in the same way by two different objective functions. The combination of one objective function (FLC) for particle picking and another (MLE) for particle alignment may allow the reconstitution of the true signal from the selected images. However, despite the application of both FLC and MLE in single-particle analysis of cryo-EM structures [22, 28–32], it remains unknown how the bi-objective function (BOF) scheme performs in terms of various control parameters, such as signal-to-noise ratio (SNR) and initialization inputs.

Beyond FLC and MLE, several machine-learning approaches, such as deep learning based on convolutional neural networks, have been applied to address the problem of signal detection in cryo-EM data [20, 33–36]. These approaches not only relieve the burden of post-picking manual selection [20, 33], but also work in a template-free fashion [34–36]. However, these advantages come at a significant computational cost. Thus, except for a few cases dealing with highly dynamic complex machineries that have benefitted from the deep-learning-based particle selection approach [37–39], most high-resolution cryo-EM structures published to date have relied heavily on FLC-based particle picking [40–42].

In the present study, we systematically evaluated how the performance of the BOF approach is affected by three variables: (1) the SNR of the cryo-EM data, (2) the choices of the template used for particle picking, and (3) the initialization reference used in MLE alignment for signal verification. We quantitatively characterized the performance and robustness of the BOF approach with simulated micrographs exhibiting a wide range of SNRs, as well as with real-world cryo-EM data of a 173-kD glucose isomerase. We performed comparative BOF studies with different references to investigate how the adverse effect of reference dependency incurred by the use of the FLC may be suppressed by the application of the MLE initialized using a Gaussian model.

## Methods

### A brief review on objective functions used for signal alignment

Within a set of *N* single-particle images, each of which is a noisy, translated and rotated copy of the underlying 2D projection structure ***A***, the *i*th image can be represented by the equation.

1 $$ {\boldsymbol{X}}_i=R\left({\phi}_i\right)\boldsymbol{A}+{\sigma \boldsymbol{G}}_i,\kern1em i=1,2,\dots N, $$

where ***X***_*i*_ is the observed *i*th image comprising *J* pixels with values *X*_*ij*_; *R*(*ϕ*_*i*_) denotes the in-plane transformation depending on the parameter vector ***ϕ***_*i*_ = (*α*_*i*_, *x*_*i*_, *y*_*i*_) that comprises a rotation *α*_*i*_ and two translations *x*_*i*_ and *y*_*i*_ along two orthogonal directions; ***A*** is the underlying signal with pixel values *A*_*j*_ that is common to all images; ***G***_*i*_ is the noise of a Gaussian distribution with a unity standard deviation, further scaled by a scalar factor *σ*. Because the parameter vector ***ϕ***_*i*_ is experimentally unknown, the problem of image alignment is to determine the solution of a set of parameter vectors **Φ** = { ${\phi}_i^{(n)}$; *i* = 1, 2, … *N*} that allows an optimal estimate of the underlying true signal through averaging of these images.

2 $$ {\boldsymbol{A}}^{\left(n+1\right)}=\frac{1}{N}{\sum}_{i=1}^N{R}^{-1}\left({\varPhi}_i^{(n)}\right){\boldsymbol{X}}_i $$

in which ${R}^{-1}\left({\phi}_i^{(n)}\right)$ is the reverse transformation that brings the image ***X***_*i*_ to the common orientation and position of ***A***. This image alignment problem may be mathematically translated into different optimization problems. Two main types of mathematical translations have emerged in past studies [24, 43]. In the first type, the image alignment problem was addressed by maximizing the squared magnitude of the summed images [43], which can be described as

3 $$ L\left(\mathbf{X},\boldsymbol{\Phi} \right)={\left\Vert {\sum}_{i=1}^N{R}^{-1}\left({\phi}_i\right){\boldsymbol{X}}_i\right\Vert}^2 $$

The maximum of this function is equivalent to the minimization of the least squares target

4 $$ {L}^{\prime}\left(\mathbf{X},\boldsymbol{\Phi} \right)={\sum}_{i=1}^N{\left\Vert {\boldsymbol{X}}_i-R\left({\phi}_i\right)\boldsymbol{A}\right\Vert}^2 $$

A local minimization of this function can be obtained by iteratively maximizing the cross-correlation between each image and the average.

5 $$ {\varPhi}_i^{\left(n+1\right)}=\arg {\max}_{\phi}\left[{\boldsymbol{X}}_i\cdot R\left({\varPhi}_i\right){\boldsymbol{A}}^{(n)}\right],i=1,2\dots N $$

Here, the dot indicates an inner product between two images $\boldsymbol{X}\cdot \boldsymbol{A}={\sum}_{k=1}^J{x}_k{a}_k$. An approximate solution may be obtained by iteratively estimating the underlying signal ***A***^(*n*)^ and the alignment parameter ${\phi}_i^{(n)}$ according to eqs. (3) and (5).

In the second type, the image alignment problem is interpreted as a maximum-likelihood estimate (MLE) of the signal ***A***, i.e. the maximization of the probability function

6 $$ \mathcal{L}\left(\varTheta \right)={\prod}_{i=1}^NP\left({\boldsymbol{X}}_i|\varTheta \right) $$

whereby *P*(***X***_*i*_| *Θ*) is the probability density function observed for the image *X*_*i*_ given the set of model parameters *Θ* = (***A***, *σ*, *ξ*), where *ξ* characterizes the statistics of *R*(*ϕ*_*i*_). In this case, the alignment parameters **Φ** = { *ϕ*_*i*_; *i* = 1, 2, … *N*} are treated as latent variables. The maximization of the probability function $\mathcal{L}\left(\varTheta \right)$ is more conveniently replaced by its logarithm

7 $$ L\left(\varTheta \right)={\sum}_{i=1}^N\ln P\left({\boldsymbol{X}}_i|\varTheta \right)={\sum}_{i=1}^N\ln \int P\left({\boldsymbol{X}}_i|\phi, \varTheta \right)P\left(\phi |\varTheta \right) d\phi $$

A local maximum of the log-likelihood function *L*(*Θ*) can be obtained by finding the value of *Θ* at which the partial derivatives of *L*(*Θ*) are zero. The problem of finding the maximum likelihood can be numerically tackled through the expectation-maximization algorithm. This algorithm is an iterative method that alternates between an expectation (E) step, which computes the expectation of the log-likelihood evaluated using the current estimate for the model parameters, and a maximization (M) step, which computes model parameters maximizing the expected log-likelihood found in the E-step [24]. These estimates of parameters are then used to determine the distribution of the latent variables in the next E-step. In each E-step, the observed data ***X***_*i*_ and the current estimates of model parameters *Θ*^(*n*)^ are used to calculate the expectation of the log-likelihood function as

8 $$ Q\left(\varTheta, {\varTheta}^{(n)}\right)={E}_{\Phi \left|X,\Theta\ \right.}\left[{\sum}_{i=1}^N\ln P\left({\boldsymbol{X}}_i,\phi |\varTheta \right)\right]={\sum}_{i=1}^N\int P\left(\phi |{\boldsymbol{X}}_i,{\varTheta}^{(n)}\right)\ln \left\{P\left({\boldsymbol{X}}_i|\phi, \varTheta \right)P\left(\phi |\varTheta \right)\right\} d\phi $$

Under the assumption of a Gaussian distribution of the latent variables **Φ** = { *ϕ*_*i*_; *i* = 1, 2, … *N*} and the observed signal, this gives rise to

9 $$ Q\left(\varTheta, {\varTheta}^{(n)}\right)\propto {\sum}_{i=1}^N\int P\left(\phi |{\boldsymbol{X}}_i,{\varTheta}^{(n)}\right)\left\{-\frac{1}{2{\sigma}^2}{\left\Vert {\boldsymbol{X}}_i-R\left(\phi \right)\boldsymbol{A}\right\Vert}^2\right\} d\phi $$

In the M-step, *Q*(*Θ*, *Θ*^(*n*)^) is maximized with respect to the model parameters

10 $$ {\varTheta}^{\left(n+1\right)}=\arg {\max}_{\varTheta }Q\left(\varTheta, {\varTheta}^{(n)}\right) $$

which corresponds to the minimization of a weighted least-squares target with a weight of *P*(*ϕ*| ***X***_*i*_, *Θ*^(*n*)^) for each image. Note that this is in marked contrast to eq. (4). The estimate of the signal therefore is a weighted average including contributions from all possible values of *ϕ* for every image ***X***_*i*_, so that the class averages can be updated in a probability-weighted manner

11 $$ {\boldsymbol{A}}^{\left(n+1\right)}=\frac{1}{N}{\sum}_{i=1}^N\int P\left(\phi |{\boldsymbol{X}}_i,{\varTheta}^{(n)}\right){R}^{-1}\left(\phi \right){\boldsymbol{X}}_i d\phi $$

All other model parameters in *Θ*^(*n* + 1)^ are updated in the M-step similarly as probability-weighted averages [24].

It is also necessary to consider the mathematical relationships and differences between the image alignment approaches. First, in recovering the signal ***A***, the latter approach uses a probability-weighted average instead of the deterministic average used in the former approach, as illustrated by the differences between eqs. (2) and (11). Second, if one assumes that the estimate of the hidden variable **Φ** is deterministic instead of probabilistic, *P*(*ϕ*_*i*_| ***X***_*i*_, *Θ*^(*n*)^) adopts the form of a Dirac δ-function. Under this condition, the maximization of the log-likelihood function shown in eq. (9) is simplified to the minimization of the least-squares target shown in expression (5), instead of the probability-weighted least-squares target in eq. (9). At the same time, the estimate of the signal by eq. (11) can be reduced to eq. (2). Third, despite this conditional equivalence in terms of numerical optimization, the two approaches adopt essentially different objective functions that include different variables and parameters, as evidenced by a comparison of eqs. (5) and (8). Importantly, all model parameters *Θ* = (***A***, *σ*, *ξ*) are re-estimated during each iteration of optimization in the latter approach, whereas only one type of model parameter, ***A***, is re-estimated during the course of optimization in the former approach.

Previously proposed solutions to the particle-picking problem were mostly derived from the cross-correlation-based approach. In a typical case, the locally normalized correlation function is calculated between a search object ***S*** (template) and target micrograph ***T*** under the footprint of a mask ***M*** [8]:

12 $$ {C}_L(x)=\frac{1}{P}{\sum}_{k=1}^J\frac{\left({S}_k-\overline{S}\right){M}_k\left({T}_{k+x}-\overline{T}\right)}{\sigma_S{\sigma}_{MT}(x)} $$

where $\overline{S}$ and *σ*_*S*_ are the average and standard deviation of the search object *S*_*k*_; $\overline{T}$, and *σ*_*MT*_ are the local average and standard deviation of ***T*** within the footprint of mask ***M***; *x* is the position of the footprint of mask ***M***, and *P* is the total number of non-zero points inside the mask. If $\overline{S}$ and *σ*_*S*_ are set to zero and unity, respectively, eq. (12) is reduced to

13 $$ {C}_L(x)=\frac{1}{P{\sigma}_{MT}(x)}{\sum}_{k=1}^J{S}_k{M}_k{T}_{k+x} $$

The local standard deviation of ***T*** can be calculated via

14 $$ {\sigma}_{MT}^2(x)=\frac{1}{P}{\sum}_{k=1}^J{M}_k{T}_{k-x}^2-{\left[\frac{1}{P}{\sum}_{k=1}^J{M}_k{T}_{k-x}\right]}^2 $$

This and other similar implementations of a particle-picking strategy have been collectively referred to as “template matching”. As the image size of ***S*** is much smaller than that of ***T***, the local cross-correlation is calculated with the mask ***M*** raster-scanning across the entire micrograph to produce a cross-correlation map. The local maximum in the correlation map is identified, ranked, and used to indicate the position of the picked candidate particle image. The FLC function expressed in eq. (13) has led to a more efficient implementation of a computational particle-picking procedure [8, 12, 13].

As explained above, the FLC function is notably different from the MLE in signal recognition in their mathematical forms. In the absence of noise, the cross-correlation function and MLE should both lead to the same solution for the image alignment problem [24]. However, in the presence of noise, the FLC and MLE behave differently [24]. The FLC is very fast and efficient in computation. However, it demonstrates an increasing propensity to identify false-positive particles or introduce mis-alignment as the SNR decreases [8, 12, 13]. By contrast, at the expense of significantly more computational power, the exhaustive probability search across parameter space in the MLE substantially reduces the effect of false positives over the iterations of the expectation-maximization algorithm. The probability-weighted averages further limit the contribution of false positives and mis-alignment to the estimation of the signal. Therefore, the FLC and MLE are complementary to each other in their responses to noise, as well as in their computational efficiency.

### Procedure of the BOF approach

**Fig. 1:**
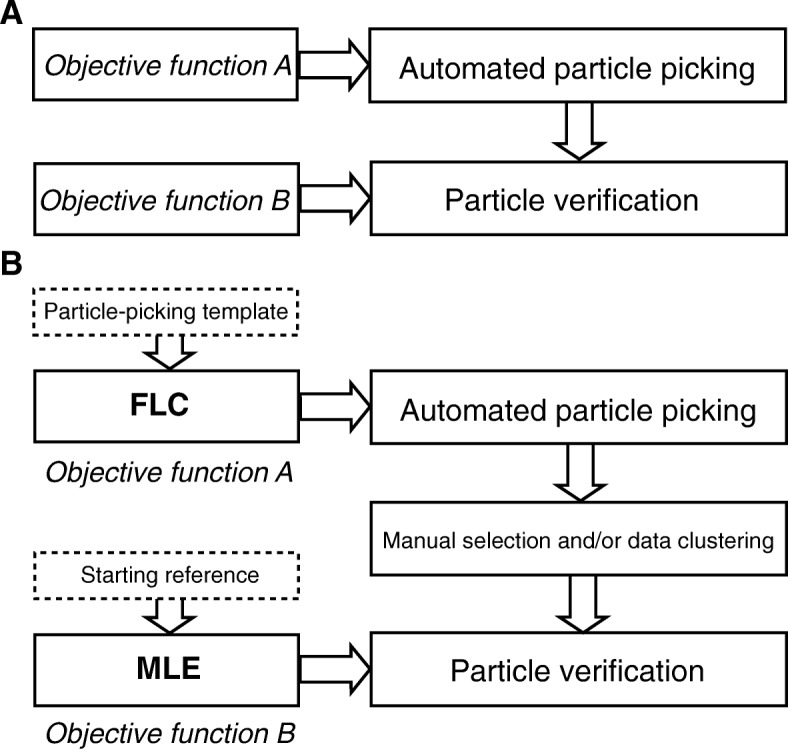
Strategy and implementation of the BOF approach. **a** The BOF approach involves the use of two different objective functions. The first objective function deals with particle detection and the second one with particle verification. **b** The BOF approach used in this study combines FLC and MLE objective functions, which are not mathematically equivalent or correlated. User-determined templates/references are shown in the dashed boxes, designated with the nomenclature used throughout this manuscript

Throughout this study, the following BOF-based procedure was applied to 26 datasets of either pure noise or simulated micrographs of the trimeric ectodomain of the influenza hemagglutinin (HA) glycoprotein [44], as well as an experimental dataset of focal-pair micrographs of the 173-kDa glucose isomerase complex. The BOF strategy and an implementation of the BOF procedure are shown in Fig. 1, a and b, respectively.

#### Step 1: Particle picking by fast local cross-correlation

We used template matching by FLC implemented in SPIDER to pick particles [45]. The SPIDER system is a comprehensive software package for image processing that supports rapid scripting to handle batch processing of cryo-EM data [45]. The SPIDER script lfc_pick.spi has already been applied to the ribosome [12] and has served as a control for the recent development of a reference-free particle-picking approach [35]. This procedure applies the FLC function to particle recognition [8]. In this study, we picked particles using single 2D templates, as described in the specific experiments below. Note that previous studies have shown that using the FLC function with a single template can pick many views of particles [12]. Nonetheless, it has been suggested that using more templates can potentially reduce the number of false positives that are picked [8, 12, 13].

#### Step 2: Candidate particle selection using a threshold in the ranking of correlation peaks and manual rejection of obvious artifacts

The SPIDER particle-picking program lfc_pick.spi sorts and ranks the picked particles according to their correlation peaks, from high to low peak values. Upon sorting and ranking, the potential true particles often appear at higher correlation peak values and the pure noise images at lower correlation peaks. A threshold that approximately demarcates the boundary between the potential true particles and pure noise can be used to select the initial candidate particles, followed by manual inspection of each particle and rejection of obvious artifacts. The rejection of suspected artifacts and false positives can be done in batch mode if the picked particles are grouped into many 2D classes by multivariate statistical analysis or unsupervised clustering [15, 19, 46, 47].

#### Step 3: Particle validation by a MLE alignment with multiple classes

Image similarity measured via the MLE-based probability, and the subsequently calculated class averages obtained by integrating over all probabilities, are more sensitive to the presence of true signals [24]. The particles belonging to the class averages that clearly exhibit the expected signal features are chosen for further processing; the particles in the class averages that are suspicious or apparently artefactual may then be discarded. This step provides an opportune checkpoint to efficiently remove non-particles in batch mode.

### BOF testing of simulated and experimental noise micrographs

To conduct a baseline control, we first simulated 200 micrographs containing only Gaussian noise using the SPIDER command MO (option R with Gaussian distribution). Each micrograph had dimensions of 4096 × 4096 pixels. We then used one projection view of the ~ 11-Å human immunodeficiency virus (HIV-1) envelope glycoprotein (Env) trimer [28] as a template for particle picking from the simulated Gaussian-noise micrographs. The box size was 256 × 256 pixels. Although the micrographs can be binned twice or 4 times to speed up the computational procedure of particle picking by FLC, it is necessary to extract the particles from unbinned original micrographs because they are required for high-resolution 3D reconstruction in later steps in an actual scenario of structure determination [48]. In each micrograph, about 20–25 boxed images of the highest local correlation peaks were selected to assemble a particle stack of 4485 images. After particle picking and selection, each particle image was scaled 4 times to 64 × 64 pixels using xmipp_scale, and normalized using xmipp_normalize [49]. Subsequent MLE alignment using xmipp_ml_align2d was repeated with three different starting references: (1) a noise image randomly chosen from the entire image stack, which contains weak signal that is likely to introduce some initiation bias; (2) a Gaussian circle, which follows a Gaussian distribution in radial intensity and does not introduce any prior bias to the reference; and (3) an average of a random subset of the unaligned images that replicates the template used for particle picking, which can be used to test the reference dependency of the MLE alignment. Comparison among these three cases would allow us to examine whether and how the initial reference used for MLE impacts the potential capability of MLE to suppress reference dependency introduced during FLC-based particle picking.

**Fig. 2:**
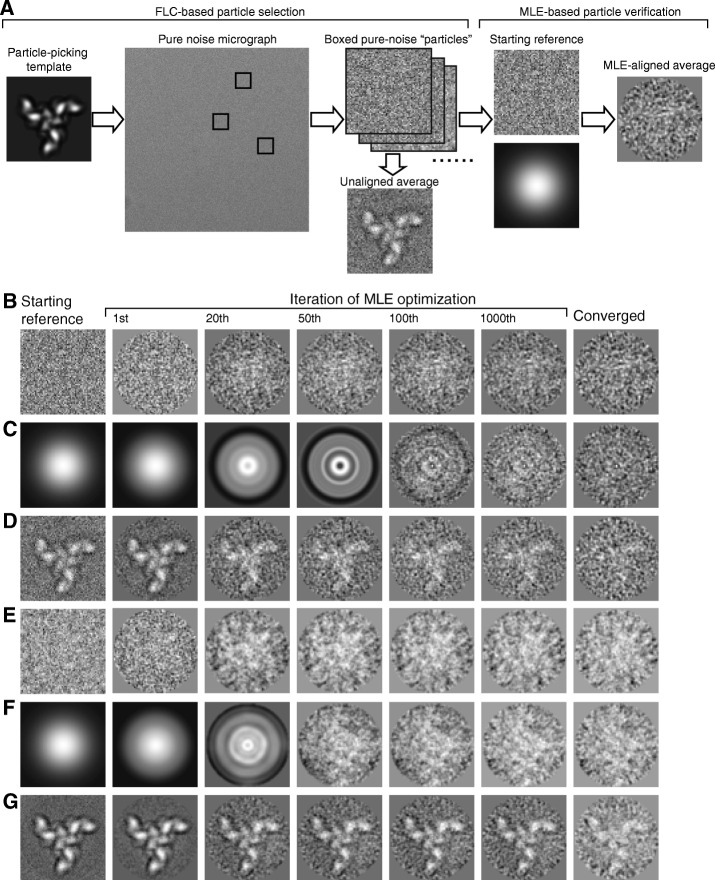
The BOF results for simulated and experimental pure noise data. **a** A schematic flow diagram showing that “particles” were picked by FLC from pure-noise micrographs, using a single projection of the HIV-1 envelope glycoprotein (Env) trimer as a template. The picked particles were subjected to MLE alignment, using different starting references. **b**-**d** The FLC-picked particle set, derived from the simulated Gaussian-noise micrographs was aligned by MLE, starting from a noise image randomly chosen from the particle set (**b**), a Gaussian circle (**c**), or the average of the picked particles (**d**). The starting reference for MLE optimization is shown in the first column. Each row shows the history of the MLE-aligned class averages at the indicated iterations of optimization, ending with the respective converged class averages in the far-right column. **e**-**g** The FLC-picked particle set derived from the experimental ice-noise micrographs and aligned using MLE, starting from a noise image randomly chosen from the particle set (**e)**, a Gaussian circle (**f**), or the average of the picked particles (**g**). The averages shown in (**d**) and (**g**) appear as an FLC-generated replica of the 2D template used for particle picking

To repeat the above BOF test on real-world experimental ice noise, we imaged a cryo-grid that was flash-frozen from a buffer containing no protein sample. The composition of the buffer was 20 mM Tris-HCl, pH 7.4, 300 mM NaCl and 0.01% Cymal-6 (Anatrace, USA). This was the same buffer used for vitrifying the HIV-1 Env trimer for its cryo-EM structural analysis [28, 32]. The cryo-grid was made from a C-flat holey carbon grid using the FEI Vitrobot Mark IV (Thermo Fisher Scientific, USA). The data were collected on an FEI Tecnai G2 F20 microscope (Thermo Fisher Scientific, USA) operating at 120 kV, equipped with a Gatan Ultrascan 4096 × 4096-pixel CCD camera (Gatan, USA), at a nominal magnification of 80,000×. We selected 218 micrographs of pure ice noise collected in one cryo-EM session. The same particle-picking procedure performed with the simulated Gaussian noise micrographs (see above) was applied to the experimental ice noise micrographs, with the same HIV-1 Env trimer template. After particle picking, the apparent ice-crystal contaminants were manually rejected from the particle set, leaving only images of amorphous ice noise. By selecting only about 10–25 boxed images with the highest local correlation peaks from each micrograph, a particle stack of 4591 images was assembled, and was subjected to the same MLE alignment as described above for the data from the simulated Gaussian noise micrographs. These BOF tests on both the simulated and experimental pure noise micrographs (Fig. 2) served as controls for the subsequent examination of the BOF approach.

### BOF testing of simulated micrographs

Throughout this study, the SNR was defined as the ratio of signal variance to noise variance [3, 50],

15 $$ \mathrm{SNR}={\sigma}_{Signal}^2/{\sigma}_{Noise}^2 $$

When the background noise has a mean value of zero, its power *P*_Noise_ equals its variance ${\sigma}_{Noise}^2$. In single-particle cryo-EM images, the particles are located at different positions in the micrographs and carry the signal. When the mean value of the signal is normalized to zero, *P*_Signal_ becomes equal to ${\sigma}_{Signal}^2$, and the power ratio of signal to noise thus equals the variance ratio. The SNR of a micrograph was calculated as the power ratio of the signal from all the particles to the background noise in this micrograph. For the SNR of a single-particle image, the noise variance was calculated on a boxed background area without any particle, and the signal variance was calculated on the particle image of the same box size without background noise.

**Fig. 3:**
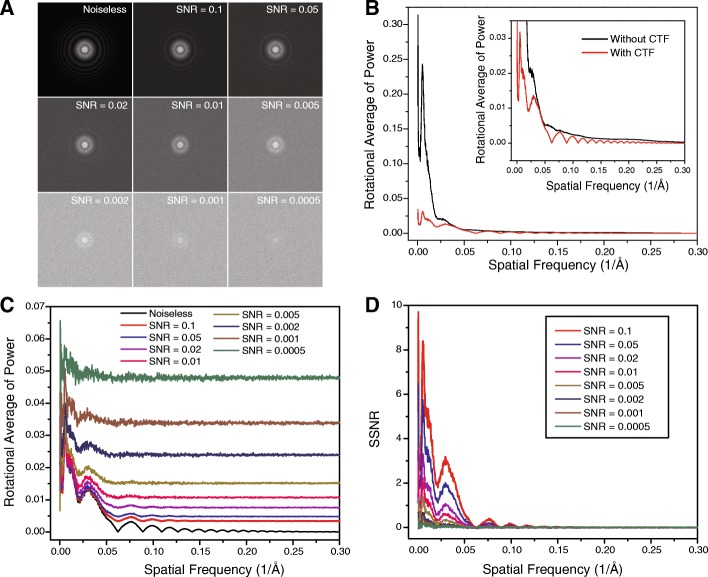
The Fourier behavior of the simulated micrographs. **a** The power spectra of the simulated micrographs with different SNRs. **b** The rotational averages of the power spectrum of the noiseless micrograph before and after applying the CTF effect. **c** The rotational averages of the power spectra of the simulated noisy micrographs. **d** The spectral signal-to-noise ratios (SSNRs) of the simulated noisy micrographs

We simulated 120 micrographs of noiseless particles corresponding to the crystal structure of the influenza A virus hemagglutinin (HA) glycoprotein ectodomain (PDB ID: 3HMG) using xmipp_phantom_create_micrograph [44]. The simulation assumed a pixel size of 1.0 Angstrom and micrograph dimensions of 4096 × 4096 pixels. To simulate the aberration effect of the objective lens in electron microscopy, the contrast transfer function (CTF) was applied in the Fourier transform of the simulated noiseless micrographs using a separate SPIDER script. The CTF simulation assumed an acceleration voltage of 200 kV, a defocus of − 1 μm, a spherical aberration Cs of 2.0 mm, an amplitude contrast ratio of 10%, and a Gaussian envelope half width of 0.333 Å^− 1^. In each simulated micrograph, there were 323 HA molecules that assumed random orientations. To add different levels of Gaussian noise to the noiseless micrographs, the standard deviation of the background of each micrograph was calculated and used as input to simulate a background Gaussian noise image that was added to the noiseless micrographs. The simulated micrographs with Gaussian noise additively yielded SNRs of 0.1, 0.05, 0.02, 0.01, 0.005, 0.002, 0.001 or 0.0005. A typical series comprising a simulated noiseless micrograph and the derived noisy micrographs at different SNRs is shown in Additional file 1: Figure S1. A comparison of the corresponding behaviors of the power spectra in Fourier space is shown in Fig. 3. Note that the SNR calculated for an entire micrograph is often lower than the SNR calculated from boxed single-particle images, since there are more empty background areas in the micrograph than in appropriately boxed single-particle images.

**Fig. 4:**
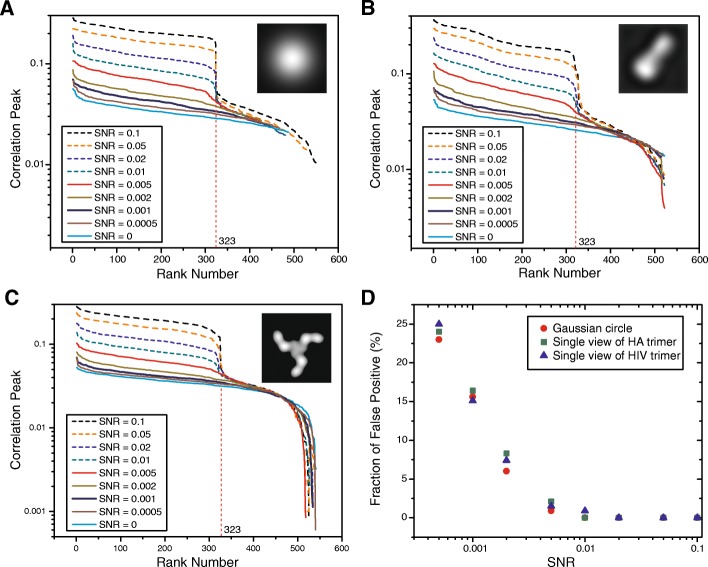
The correlation-peak ranking plots and differentiation of true-positive and false-positive particles in FLC-based automated particle picking. The correlation-peak ranking plots corresponding to different SNRs, obtained using three different particle-picking templates: (**a**) a Gaussian circle, (**b**) one projection view of the influenza virus HA trimer, and (**c**) one projection view of the HIV-1 Env trimer. The particle-picking templates are shown in the insets. All plots are from the noisy particle micrographs derived from the same simulated noiseless micrograph of the influenza virus HA trimer. Note that the position of the drop-off in the correlation peak values corresponds to 323, which was the number of actual influenza virus HA trimers in the simulated micrographs. (**d**) Rate of false positivity in particle picking. The plots of false positive fraction against SNR in particle picking using the three different templates are shown, indicating that the specificity of FLC particle picking is highly dependent on the SNR, and is also affected to a lesser extent by the choice of the 2D template. Below a critical SNR range (0.002–0.005), the percentage of false positives rises considerably

**Fig. 5:**
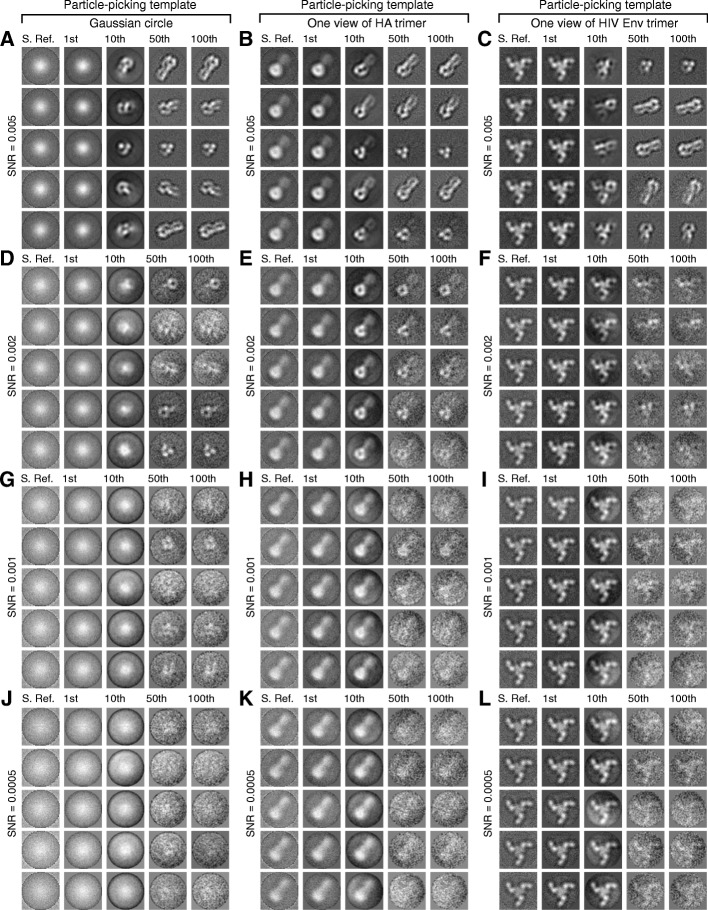
Effects of the particle-picking template used in FLC and the micrographs’ SNR on MLE optimization. Noisy micrographs showing influenza virus HA trimers with different SNRs were subjected to BOF testing, using different templates for particle picking. The corresponding SNRs of the micrographs from which the particle sets were picked were 0.005 (**a**, **b** and **c**), 0.002 (**d**, **e** and **f**), 0.001 (**g**, **h** and **i**) and 0.0005 (**j**, **k** and **l**). The templates used for particle picking were: a Gaussian circle (**a**, **d**, **g** and **j**), one projection view of the influenza virus HA trimer (**b**, **e**, **h** and **k**) and one projection view of the HIV-1 Env trimer (**c**, **f**, **i** and **l**). The particles picked by FLC were randomly divided into five classes and averaged. The resulting “class averages” are shown in the leftmost column of each panel (**a**-**l**). Each assembly of datasets was subjected to multi-reference MLE classification using the random class averages as starting references. In each panel, the five rows of image series correspond to five particle orientation classes generated by MLE, with the starting reference (S. Ref) and class averages of the milestone iterations (1st, 10th, 50th, and 100th) shown in a row. The BOF testing results show that MLE optimization can recover the weak signal of the influenza virus HA trimer if the images have a sufficiently high SNR

**Fig. 6:**
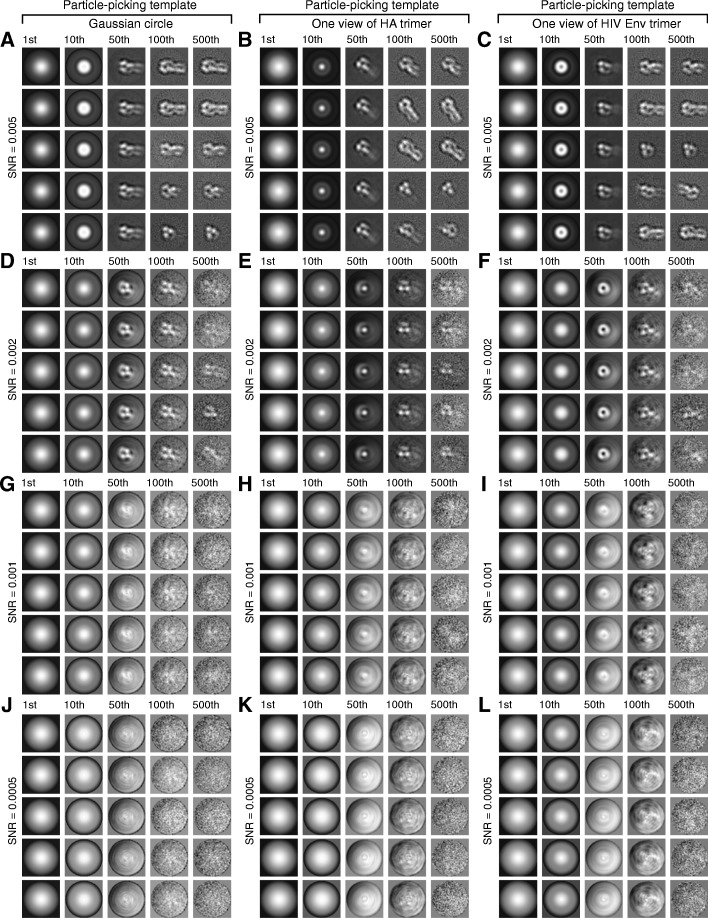
Effects of using a Gaussian circle as the starting reference for MLE optimization. The procedures shown in Fig. 5 were repeated with a Gaussian circle as the starting reference for all the data sets in the multi-reference MLE classification. The corresponding SNRs of the micrographs from which the particle sets were picked were 0.005 (**a**, **b** and **c**), 0.002 (**d**, **e** and **f**), 0.001 (**g**, **h** and **i**) and 0.0005 (**j**, **k** and **l**). The templates used for particle picking were: a Gaussian circle (**a**, **d**, **g** and **j**), one projection view of the influenza virus HA trimer (**b**, **e**, **h** and **k**), and one projection view of the HIV-1 Env trimer (**c**, **f**, **i** and **l**). In each panel, the five rows of image series correspond to five particle orientation classes generated by MLE, with the class averages of the milestone iterations (1st, 10th, 50th, 100th, 500th) shown in a row. At an SNR of 0.002 and above, the particle-picking template is not recapitulated by the MLE optimization when a Gaussian circle is used as the starting reference

For the simulated micrographs at each SNR value, we conducted BOF tests using three different templates for particle picking: a Gaussian circle, one projection view of the influenza virus HA trimer filtered to 30 Angstroms, and one projection view of the HIV-1 Env trimer filtered to 30 Angstroms (Fig. 4). Each set of micrographs with a given SNR and selected by a particular particle-picking template was treated as a separate case. Therefore, there were 8 × 3 = 24 cases studied and compared in our BOF tests. For each case, a stack of 38,760 particle images was assembled from 120 simulated micrographs, based on a selection threshold of 323 particles per micrograph. The original box dimension for particle picking was 180 × 180 pixels. After particle picking and selection, each particle image was first scaled 3 times to a dimension of 60 × 60 pixels, normalized for background noise, and subjected to multi-reference MLE classification into 5 classes, using two different initial references: (1) the average of a randomly selected subset of particles (Fig. 5), and (2) a Gaussian circle, which follows a Gaussian distribution in radial intensity (Fig. 6). When extrapolating to the SNR of single-particle images, the SNR of an entire micrograph needs to be multiplied by a factor (> 1), which depends on the particle density and the box size of particles, to make it equivalent to the SNR of single-particle images. Given the aforementioned parameters, the SNRs of the simulated micrographs at 0.1, 0.05, 0.02, 0.01, 0.005, 0.002, 0.001 and 0.0005 correspond to the single-particle SNRs of 0.16, 0.08, 0.032, 0.016, 0.008, 0.0032, 0.0016 and 0.0008, respectively. Throughout the rest of this paper, unless stated explicitly, the “SNR” refers to that of the simulated micrographs instead of the single-particle SNRs.

### BOF tests on experimental cryo-EM data

We collected an experimental cryo-EM dataset of the 173-kDa glucose isomerase complex (Hampton Research, CA, USA). A 2.5-μl drop of a 3 mg/ml glucose isomerase solution was applied to a glow-discharged C-flat grid (R 1.2/1.3, 400 Mesh, Protochips, CA, USA), and flash-frozen in liquid ethane using the FEI Vitrobot Mark IV (Thermo Fisher Scientific, USA). The cryo-grid was imaged in an FEI Tecnai Arctica microscope (Thermo Fisher Scientific, USA) at a nominal magnification of 21,000× and an acceleration voltage of 200 keV. We selected 95 focal pairs of micrographs collected using a Gatan K2 Summit direct detector camera (Gatan Inc., CA, USA), with a defocus difference of 1.5 μm and a pixel size of 1.74 Å. The actual defocus values of the micrographs were determined through CTFFind3 [51]. The first exposure was taken at a defocus between − 1.0 and − 3.0 μm. In this defocus range, the visibility of the complexes was marginal, posing difficulties for manual particle identification. The second exposure was taken at a defocus between − 3.0 and − 5.0 μm. In this defocus range, the particles were more visible. We then used FLC to pick particles directly from the micrographs of the first exposure, and used the second exposure to manually verify the particle selection from the first exposure. Using the first exposure at a lower defocus, which gives lower single-particle SNRs, provides a more stringent test of the robustness of the BOF approach than using the second exposure at a higher defocus.

To perform BOF tests on these cryo-EM data, we assembled three particle stacks (comprising 22,298, 20,632 and 22,828 particles, respectively) using three different templates for particle picking, i.e., a Gaussian circle, one projection view of the glucose isomerase crystal structure (PDB ID: 1OAD) filtered to 30 Å, and one projection view of the HIV-1 Env trimer filtered to 30 Å. Particle images of 90 × 90 pixels, picked by FLC, were phase-flipped to partially correct the CTF effect. The three stacks of particles were normalized for background noise and subjected to multi-reference MLE classification into 5 classes, using two different initial references: (1) the average of a randomly selected subset of particles; and (2) a Gaussian circle, which follows a Gaussian distribution in radial intensity.

## Results

### BOF tests on simulated and experimental noise

As a control experiment to investigate the ability of the BOF approach to resist reference bias, we conducted BOF tests on simulated micrographs that contain only Gaussian noise. A single 2D projection of the HIV-1 Env trimer was used as the template for picking “particles” by FLC (Objective function A) (Fig. 2a). Images with the highest local correlation peaks were selected and subjected to MLE alignment, using three different starting references for MLE optimization (Objective function B). In the first BOF test, a raw pure noise image randomly chosen from the particle stack was used as the starting reference for MLE optimization (Fig. 2b). Over more than 3000 iterations of MLE alignment, no 2D structure resembling the particle-picking template was observed. The resulting average image in each iteration was still a random noise image. We then used a Gaussian circle as the starting reference to repeat the MLE optimization (Fig. 2c). Again, the resulting average image contained only random noise but no observable 2D model. As the third starting reference for MLE optimization, we used the average of template-selected particle images without any further alignment. Notably, this average closely resembled the HIV-1 Env trimer template used for particle picking (Fig. 2d), and apparently resulted from reference dependency in template-based particle picking by the FLC. When this average image was used as the starting reference for the MLE alignment, the replica of the template faded away in the average image and nearly disappeared upon the convergence of MLE optimization. Thus, the BOF approach can work against reference bias associated with the alignment of pure noise during the particle-picking process, particularly when the MLE verification is conducted using a random noise image or a Gaussian circle as the starting reference. Note that in the above-mentioned test, we performed up to 3000 iterations of MLE optimization. Such a prolonged optimization provides the computation with a greater opportunity to evade local optima and helps to examine the robustness of the convergence [24].

Next, we wanted to know if the results observed with the simulated micrographs of Gaussian noise would be reproduced with images of actual cryo-EM noise resulting from amorphous ice. We repeated the BOF tests on the dataset assembled from experimental ice noise micrographs. When aligned using MLE, starting with pure noise or a Gaussian circle as the starting reference, no structure was observed after more than 3000 iterations of optimization (Fig. 2e and f). Thus, images of experimental ice noise taken by a CCD camera reproduced the results observed with simulated Gaussian noise, supporting the notion that the experimental cryo-EM noise from amorphous ice basically exhibits Gaussian-like behavior [3]. Particle verification by MLE with starting references comprising random noise or a Gaussian circle effectively removed reference bias arising from the alignment of simulated or experimental noise. By contrast, when the unaligned average of the template-selected images was used as the starting reference for MLE alignment, the structure of the particle-picking template in the class average faded over the iterations of MLE, but was not completely removed by the MLE alignment (Fig. 2g).

### FLC performance on simulated micrographs with different SNRs

We further tested the FLC-based particle-picking program on a number of simulated micrograph datasets (Additional file 1: Figure S1). As expected, the visibility of particles was drastically diminished in the images with lower SNRs [52]. Figure 3 shows the power spectra of the simulated micrographs and their corresponding spectral SNRs (SSNRs). We applied a number of contrast-enhancement techniques, including histogram normalization, contrast stretching, low-pass filtering and pixel binning, to the simulated micrographs with different SNRs. We found that these approaches were insufficient to restore unambiguous visibility to particles when the SNR approached 0.005 (Additional file 1: Figure S2). Because the loss of visibility created difficulties with directly verifying the true and false positives in the same micrograph in our particle-picking test, the original noiseless micrograph from which the low-contrast micrograph was derived was used to verify the particle-picking performance (Additional file 1: Figure S3).

Using the noisy micrographs containing the randomly oriented influenza virus HA trimers, we picked particles using three different templates -- a Gaussian circle, one projection view of the influenza virus HA trimer, and one projection view of the HIV-1 Env trimer. Figures 4a-c show the plots of the correlation peaks versus the rank numbers of the picked particles. Notably, when the Gaussian circle was used as a template (Fig. 4a), the plots corresponding to SNRs of 0.1, 0.05, 0.02 and 0.01 showed a clear-cut drop-off in the value of the correlation peak at a rank of 323, which was the number of actual simulated particles in each micrograph [4]. All of these 323 particles with high correlation peak values were confirmed to be true positives. When the Gaussian circle was used to pick particles from micrographs with an SNR of 0.005, the plot of the correlation peaks still exhibited a discernible drop-off at *N* = 323, but with a much smoother edge (Fig. 4a). The drop-offs in correlation peak values were smoother and less prominent at lower SNR values (0.002, 0.001 and 0.0005). Using 323 as the threshold for particle selection, the number of false positives was less than 2% at an SNR of 0.005, and increased to approximately 7% at an SNR of 0.002 (Fig. 4d).

We evaluated the specificity of particle picking when using templates other than a Gaussian circle, i.e., one projection view of the influenza virus HA trimer itself, and one projection view of the HIV-1 Env trimer, which bears little similarity to the HA trimer (Fig. 4b and c). For both templates, clear drop-offs in the correlation peak-ranking plots at N = 323 were observed at SNR values of 0.005 and higher. Notably, in all cases where we used different templates in the particle-picking test, the false-positive rate was below 2.5% at the SNR values of 0.005 and above; there were no false positives at SNR values of 0.02 and greater (Fig. 4d). However, using the Gaussian circle template allowed better centering of picked particles than using the other two templates (Additional file 1: Figures S3 and S4). Among the cases compared here, the centering of picked particles was the worst when a dissimilar 2D structure (the HIV-1 Env trimer) was used as a template for micrographs with the lowest SNRs (0.005–0.0005) (Additional file 1: Figure S4). This implies that particle recognition is less sensitive to the detailed shape of the particle-picking template than are the specificity and particle-centering accuracy. Thus, the use of a dissimilar template allowed overall particle recognition, but resulted in a greater miscentering of the picked particles and more false positives at the lowest SNRs (0.005–0.0005).

### BOF tests on the simulated cryo-EM datasets

We evaluated the ability of the BOF approach to verify the presence of genuine signals in the particles selected from micrographs with different SNRs using different particle-picking templates. Strikingly, for those datasets derived from micrographs with SNRs higher than 0.002, the class averages after the MLE alignment all recapitulated the projection views of the influenza virus HA trimer, no matter what type of initial reference was used for both FLC and MLE (Figs. 5 and 6). The MLE alignment results using particles selected from micrographs with SNR values of at least 0.002 were comparable for those selected using the three distinct templates. Evidently, the model used for the particle-picking template does not govern the outcome of MLE optimization when a sufficiently strong signal is present. Below the SNR value of 0.002, the MLE reduced but did not completely remove the reference dependency in the converged class averages when the unaligned class average was used as the starting reference for MLE alignment (Fig. 5i and l). Nonetheless, this effect was substantially reduced in the converged class averages when the Gaussian circle was used as the starting reference for the MLE alignment (Fig. 6i and l).

### BOF tests on experimental cryo-EM data of glucose isomerase

**Fig. 7:**
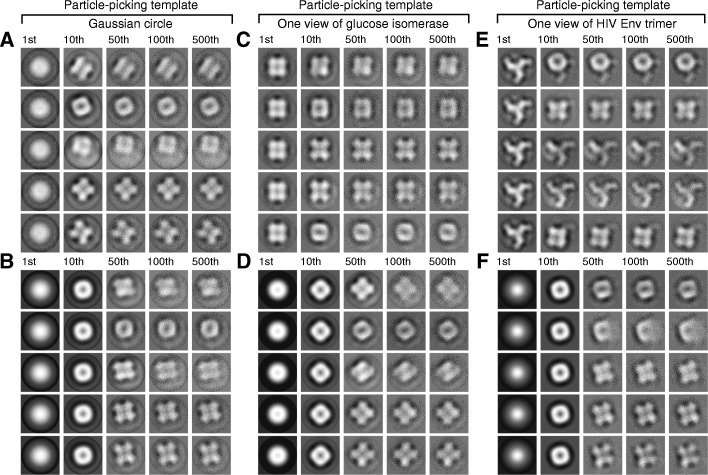
Effects of different particle-picking templates and starting references in MLE optimization of real-world cryo-EM images of the glucose isomerase complex. The templates used for particle picking were: a Gaussian circle (**a**, **b**), one projection view of the glucose isomerase complex (**c**, **d**), and one projection view of the HIV-1 Env trimer (**e**, **f**). The approximate percentages of false-positive particles assembled in the three cases, estimated through the manual examination of the larger-defocus micrographs in the focal pairs, were 6% (**a**, **b**), 4% (**c**, **d**) and 11% (**e**, **f**). In the MLE optimization step, the unaligned averages of randomly classified particles were used as starting references in panels **a**, **c** and **e**, and a Gaussian circle was used as the starting reference in panels **b**, **d** and **f**. In each panel, the five rows of image series correspond to five particle orientation classes generated by MLE, with the class averages of the milestone iterations (1st, 10th, 50th, 100th, 500th) shown in a row

To further examine the robustness of the BOF approach, we applied BOF tests to an experimental cryo-EM dataset of the 173-kDa glucose isomerase complex (Additional file 1: Figure S5). The single-particle SNR of this dataset is approximately 0.005–0.01. The BOF tests successfully produced class averages that corresponded to projection views of the glucose isomerase complex in all six cases (Fig. 7 and Additional file 1: Figure S6). Consistent with our observations with the simulated micrographs, the use of a Gaussian circle as both the particle-picking template and the MLE alignment reference performed as well or better than the other combinations in generating class averages corresponding to glucose isomerase projections (Fig. 7b). When the HIV-1 Env trimer was used as the particle-picking template and the unaligned average used as the starting reference for MLE alignment, two class averages showed structures that were strongly biased by the particle-picking template (rows 3 and 4 in Fig. 7e). By contrast, the other three class averages more closely reflected the low-resolution projection views of glucose isomerase (rows 1, 2 and 5 in Fig. 7e), although some residual elements of the HIV-1 Env trimer persisted in the background. However, when the Gaussian circle was used as the starting reference for MLE alignment, the particle-picking template of the HIV-1 Env trimer was no longer recapitulated in any of the converged class averages (Fig. 7f). Even when one of the class averages demonstrated indistinct features, perhaps due to a clustering of non-particle false positives, the aligned average did not resemble the particle-picking template of the HIV-1 Env trimer (second row in Fig. 7f). As discussed above, such classes of particles can be discarded, which provides an opportunity to cull non-particles in batch mode. These results therefore indicate that the BOF approach, when used with Gaussian references, can be successfully applied to experimental cryo-EM data of a 173-kD protein complex.

### BOF robustness

**Fig. 8:**
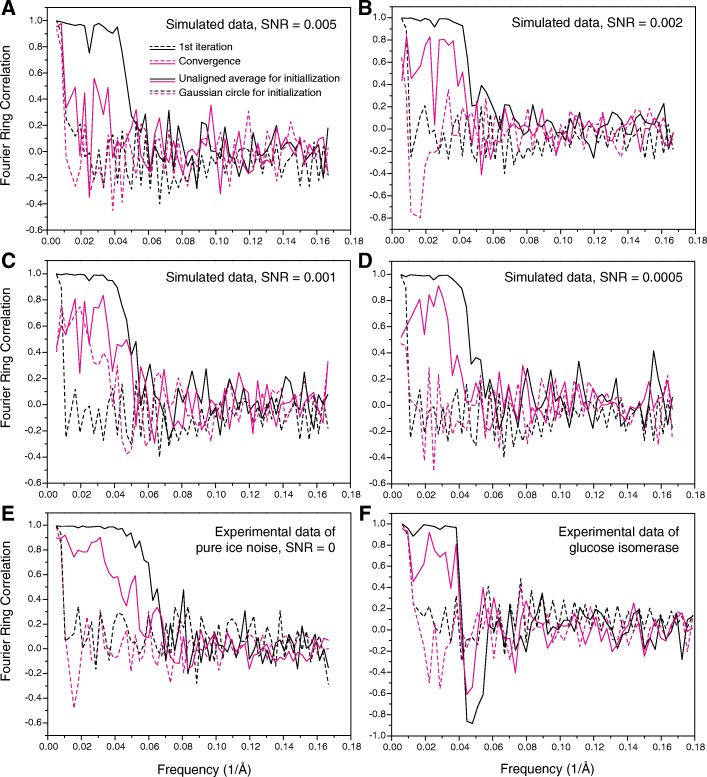
Fourier ring correlation (FRC) between the class averages and the particle-picking template of the HIV-1 Env trimer. Panels A-D show the results using the class averages of the simulated data of the HA trimer, with SNR = 0.005 (**a**), 0.002 (**b**), 0.001 (**c**) and 0.0005 (**d**), corresponding to the results shown in Fig. 5c, f, i and l and Fig. 6c, f, i and l. **e** shows the results using the pure ice noise data in the absence of any proteins, as demonstrated in Fig. 2f and g. **f** shows the results obtained with real-world cryo-EM data of the 173-kDa glucose isomerase complex, corresponding to Fig. 7e and f. The solid and dashed curves were computed from the class averages from MLE optimization using the unaligned averages and a Gaussian circle as starting reference, respectively. The color indicates the iteration of MLE optimization at which the class average was computed. For each case, the FRC analysis is shown for a single class average. The results were similar for other class averages in each case

The ability of BOF tests to suppress reference bias can be quantitatively evaluated by assessing the Fourier ring correlation (FRC) between the particle-picking template and the class averages as they evolve during the process of MLE optimization. We first analyzed the cases in which the HIV-1 Env trimer was used to pick particles, and unaligned class averages were used as starting references for MLE optimization (solid curves in Fig. 8). In these cases, the FRC curves showed a significant correlation (> 0.5) in the low-resolution range (20–50 Å) at the beginning of the MLE optimization (black solid curves in Fig. 8). However, as MLE optimization progressed to convergence, the FRC values decreased and the image of the particle-picking template diminished in significance (red solid curves in Fig. 8). In the case of the simulated data at an SNR of 0.005, the frequency of FRC-0.5 dropped to 0.015 Å^− 1^ upon convergence, indicating an efficient removal of reference bias (Fig. 8a). Correspondingly, the converged class averages efficiently recovered the projection views of the influenza virus HA trimer (Fig. 5c). At SNRs of 0.002 and lower, the frequency of FRC-0.5 was reduced to 0.02–0.04 Å^− 1^ upon convergence, indicating a less efficient removal of reference bias (Figs. 8b-e). By contrast, in all MLE alignments performed using a Gaussian circle as the starting reference, the FRC curves showed no significant correlation (> 0.5) between the particle-picking template and the converged class averages at a spatial frequency higher than ~ 0.02 Å^− 1^ (dashed curves in Fig. 8). Thus, when a Gaussian model was used as the starting reference for MLE optimization, the converged class averages did not recapitulate the structure of the particle-picking template.

## Discussion

This study provides insights into the numerical performance of the BOF procedure in the detection of weak signals. First, the FLC implementation in SPIDER successfully picked particles from micrographs with SNRs as low as 0.002–0.005, at least in our tests (Fig. 4); such low SNRs are potentially relevant to small proteins below 200 kD or certain views of larger proteins with less ordered or dynamic structures. Together with previous studies [8, 12, 13], our results suggest that the FLC function is sensitive to the presence of weak signals. A Gaussian circle seems to be as effective at picking particles as a single projection view of the imaged molecule. Second, the output parameters in the particle-picking problem are the *x*-*y* coordinates of the particle box. The choice of template in particle picking affects the coordinates of the extracted boxes, probably through biases in the correlation between the noise and the template. Consequently, the average image of the picked particles after boxing and before alignment closely resembled the particle-picking template. However, the template does not change the true signal in the boxed particle images, which allows objective signal validation by the MLE function with proper initialization. Third, the adverse effects of reference bias resulting from FLC-based particle picking can be suppressed by MLE-based alignment using a Gaussian circle as the starting reference. In other words, the reference bias derived from the FLC function does not necessarily translate into reference bias in the MLE function initialized with a Gaussian model. Finally, at the lowest SNRs (0.001 and below), the BOF procedure became inefficient at verifying signals from our dataset of 38,760 particles. In this case, the MLE alignment initialized with a Gaussian model mostly led to a blank or blurred class average that was insufficient to reproduce the particle-picking template. A similar lower bound of SNR (0.001 and below) was also found for a deep-learning-based particle-picking approach [34].

We found that the use of a dissimilar structure as the particle-picking template slightly increased the number of false positives in the examined cases. Thus, a Gaussian circle could be a preferred picking template in the initial stage of automated particle picking, since it can help avoid any potential selection bias [6]. Notwithstanding, although the Gaussian model works well for picking particle images of globular proteins or similar macromolecules, it could be error-prone and potentially miss particles with unique shapes and topologies, such as ring-like and other centrally sparse structures [6]. In this case, a validated initial model low-pass filtered at 30–60 Å, which follows the low-frequency features of the particles, could be used as a particle-picking template.

False-positive particles, such as ice contamination, can hardly be avoided by the FLC function. Nevertheless, the percentage of false positives in the candidate particle pools can be reduced by manual curation [8, 12, 13, 19]. Moreover, recent advances in applying machine learning to particle recognition can mostly remove these types of false positives, with little manual intervention [34, 35]. Thus, the objective functions in the BOF approach could be replaced with more advanced ones, such as those based on deep learning or manifold learning [34, 47], to further improve the performance of signal detection by the BOF approach.

Importantly, the aforementioned technical insights can be used to optimize and quality control the everyday practice of cryo-EM data processing. First, all current implementation of FLC-based template-matching procedures, such as those in SPIDER [45] and RELION [22], requires 2D templates derived either from 2D class averaging of thousands of manually picked particles or from 2D projections of an initial 3D model, both of which are still time-consuming and laborious to achieve. The use of a Gaussian circle as a default template for initial FLC-based particle picking can improve the level of automation and save significant labor in generating initial 2D class averages or 3D models. This strategy has already been successful in high-resolution cryo-EM structure determination in a few cases [42].

Second, in our practice of cryo-EM data processing, we have found that templates for FLC derived by averaging manually selected particles can potentially generate bias in particle picking toward the views with orientations similar to those of the templates. This is particularly a concern for smaller proteins below 200 kD or non-globular particles (plate-like, discoidal or rod-shaped, etc.) [30], of which some views might have much lower contrast or SNR than other views and could thus evade visual detection in initial manual picking. If certain views that have projection structures or shapes significantly different from the orthogonal views are missed or not included in the particle-picking templates, the FLC procedures can potentially result in more false negatives of these views, causing artificial orientation preference in the selected particle dataset. In this case, we have found that the use of a Gaussian circle as an FLC template to thoroughly pick all potential particles, followed by deeper 2D classification using statistical manifold learning [47], can reduce or avoid the artificially introduced orientation preference in the particle selection, thus eventually improving the quality and resolution of the 3D reconstruction.

Third, it has been previously hypothesized that wrong templates used for particle picking can be inadvertently recapitulated in the final 3D reconstruction of these particles, resulting in the visualization of nonexistent objects [53–55]. The present study systematically demonstrates that, given sufficient SNR in the images, such an outcome is unlikely when a Gaussian circle is used to initiate the image alignment by MLE, regardless of what type of template is used for FLC. When the initiation reference for MLE is the same as the template used for FLC on the data with lower SNRs (0.001 or lower), elements of the particle-picking template can be recapitulated in some 2D class averages generated by MLE, and could potentially bias the resulting 3D reconstruction. Thus, the use of a Gaussian circle to initialize MLE-based image alignment and refinement can be very useful for either validating the authenticity of the reconstruction or safeguarding routine cryo-EM data processing over a broad range of SNRs, avoiding the reconstruction of nonexistent structures and features out of noise [31].

Our study of the variables that affect BOF performance was limited to the combination of FLC and MLE. There are other choices for the two distinct objective functions in the BOF framework. For example, the FLC can be replaced with a deep convolutional neural network [34]. With additional testing, these modifications may further improve the utility of the BOF framework in real cryo-EM data processing pipelines.

## Conclusions

In this work, we examined the effects of SNR and choice of initialization on the ability of the BOF approach to select and verify particles from noisy cryo-EM micrographs. We quantitatively characterized the critical SNR at which BOF performance begins to degrade, and found it to be surprisingly small, as low as 0.002–0.005, given the size of the dataset (38,760 particles) tested in each case. Importantly, reference dependency of the FLC does not necessarily transfer to the MLE, making possible the robust detection and validation of weak signals. When a non-Gaussian template is used for particle picking by the FLC, the use of a Gaussian model to initialize the MLE optimization can largely suppress reference dependency of the FLC on the particle-picking template. Thus, given an SNR above the critical value, the combination of two distinct objective functions may provide a sensitive and robust way to detect and verify weak signals in cryo-EM micrographs. The essential insights into the numerical behavior of the BOF approach provided by our systematic study can guide optimization of weak signal verification and improve automation efficiency in the cryo-EM data processing pipeline for high-resolution structural determination.

## Additional file

Additional file 1: **Figure S1**. The simulated micrographs with different SNRs. **Figure S2**. Contrast enhancement of the simulated micrographs by a number of conventional techniques, including histogram normalization, contrast stretching, low-pass filtering and binning, at the SNRs of 0.005 (A) and 0.002 (B). **Figure S3**. An example of FLC-based particle picking from micrographs of the influenza virus HA trimer with low SNRs. **Figure S4**. Comparison of the FLC-based particle-picking results near the critical SNR with different templates. **Figure S5**. Automated particle picking from low-defocus (close-to-focus) micrographs and manual verification of picked particles from high-defocus (far-from-focus) micrographs. **Figure S6**. Verification of the class averages after ML classification for the BOF tests on the real cryo-EM data, using the atomic model of the glucose isomerase complex (PDB ID: 1OAD). (PDF 18817 kb)

## Abbreviations

BOF: Bi-objective function
Cryo-EM: Cryo-electron microscopy
FLC: Fast local correlation
MLE: Maximum likelihood estimate
SNR: Signal-to-noise ratio
