# Supplementary material for: Robustness of signal detection in cryo-electron microscopy via a bi-objective-function approach
Source: BMC Bioinformatics. 2019 Apr 3;20:169. doi: 10.1186/s12859-019-2714-8 (PMC6446299; doi:10.1186/s12859-019-2714-8)
Supplement: Supplementary file 1 — Figure S1. The simulated micrographs with different SNRs. Figure S2. Contrast enhancement of the simulated micrographs by a number of conventional techniques, including histogram normalization, contrast stretching, low-pass filtering and binning, at the SNRs of 0.005 (A) and 0.002 (B). Figure S3. An example of FLC-based particle picking from micrographs of the influenza virus HA trimer with low SNRs. Figure S4. Comparison of the FLC-based particle-picking results near the critical SNR with different templates. Figure S5. Automated particle picking from low-defocus (close-to-focus) micrographs and manual verification of picked particles from high-defocus (far-from-focus) micrographs. Figure S6. Verification of the class averages after ML classification for the BOF tests on the real cryo-EM data, using the atomic model of the glucose isomerase complex (PDB ID: 1OAD). (PDF 18817 kb) [file 12859_2019_2714_MOESM1_ESM.pdf]

Supplementary Material for

Robustness of signal detection in cryo-electron microscopy via a bi-objective-function approach

Wei Li Wang[1-4,§], Zhou Yu[5,§], Luis R. Castillo-Menendez[3,4], Joseph Sodroski[3,4,6], Youdong Mao[1-4,*]

[1]Intel® Parallel Computing Center for Structural Biology, Dana-Farber Cancer Institute, Boston, MA 02215. [2]Center for Quantitative Biology, School of Physics, Peking University, Beijing 100871, China. [3]Department of Cancer Immunology and Virology, Dana-Farber Cancer Institute, Boston, MA 02215. [4]Department of Microbiology and Immunobiology, Harvard Medical School, Boston, MA 02115. [5]Graduate School of Arts and Sciences, Department of Cellular and Molecular Biology, Harvard University, Cambridge, MA 02138. [6]Department of Immunology and Infectious Diseases, Harvard School of Public Health, Boston, MA 02115.

[§]These authors contributed equally to this work.

*Corresponding author. E-mail address: youdong_mao@dfci.harvard.edu (Y. M.).

Table of Contents

Supplementary Figures (2-10)

Supplementary Protocol (11-13)

Supplementary Software (14-22)

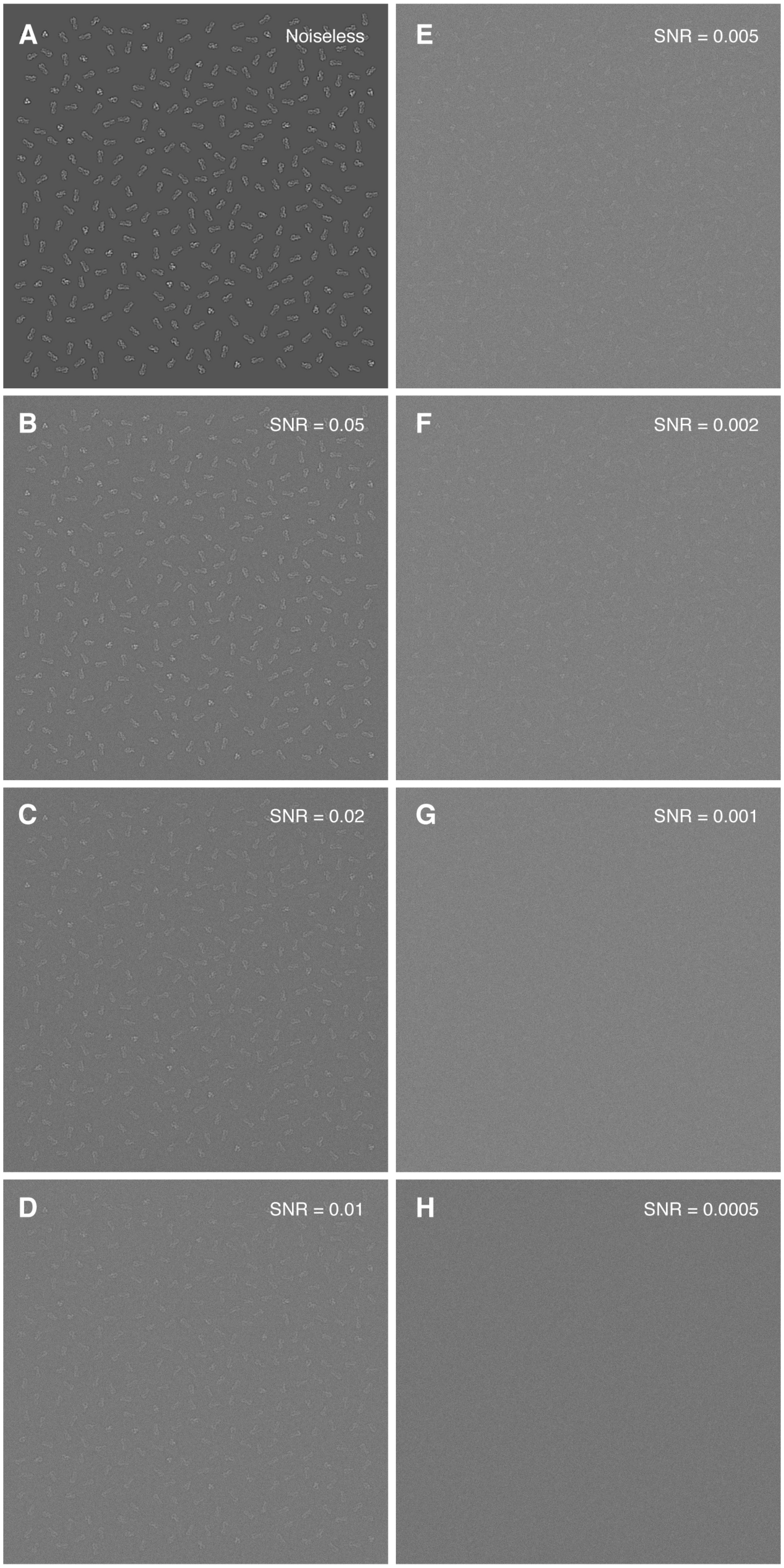
A
Noiseless
E
SNR = 0.005
B
SNR = 0.05
F
SNR = 0.002
C
SNR = 0.02
G
SNR = 0.001
D
SNR = 0.01
H
SNR = 0.0005

Figure S1. The simulated micrographs with different SNRs. (A) An example is shown of a simulated noiseless micrograph containing projection views of the influenza virus HA trimer in random orientations. (B-H) A different level of Gaussian noise was added to the noiseless micrograph shown in (A) to simulate noisy micrographs at an SNR of 0.05 (B), 0.02 (C), 0.01 (D), 0.005 (E), 0.002 (F), 0.001 (G), and 0.0005 (H).

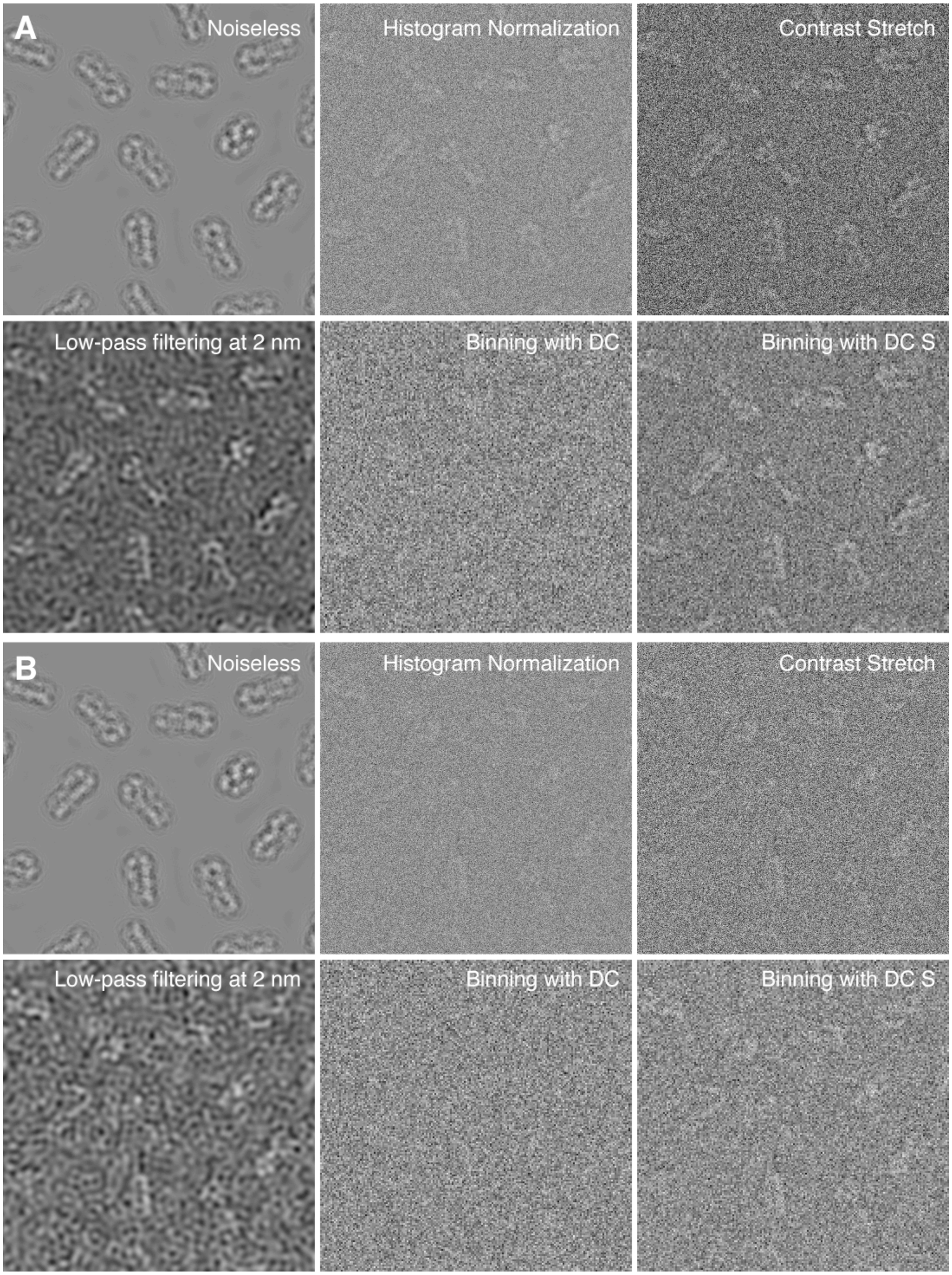

Figure S2. Contrast enhancement of the simulated micrographs by a number of conventional techniques, including histogram normalization, contrast stretching, low-pass filtering and binning, at the SNRs of 0.005 (A) and 0.002 (B). The operation of binning with SPIDER command DC results in certain loss of contrast compared to the operation of binning with SPIDER command DC S. The DC

operation bins pixels by omitting redundant pixels, whereas the DC S operation bins pixels by averaging the binned pixels.

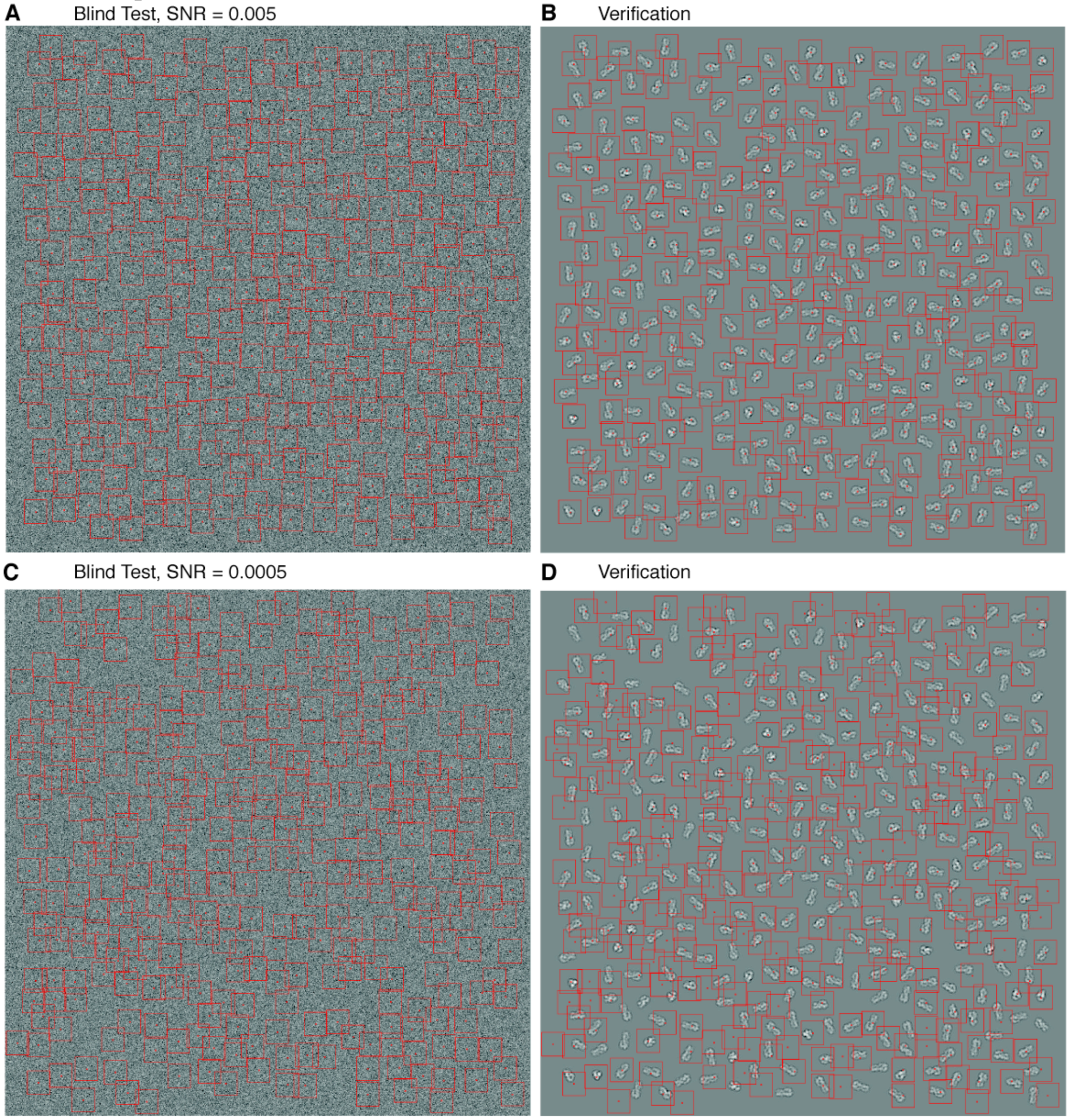

Figure S3. An example of FLC-based particle picking from micrographs of the influenza virus HA trimer with low SNRs. (A) The simulated noisy micrograph of influenza virus HA trimers at an SNR of 0.005 is shown, superposed with all 323 particle boxes (red) picked by FLC with a Gaussian circle particle-picking template. (B) The simulated noiseless micrograph that was used to derive the micrograph shown in (A), with the same 323 particle boxes (red) superposed on the micrograph. This was used for visual verification of the performance of FLC-based particle picking, showing the absence of false positives. (C) The simulated noisy micrograph of influenza virus HA trimers at an SNR of 0.0005, superposed with all 323 particle boxes (red) picked by FLC with a Gaussian circle particle-picking template. (D) Verification of the particle-picking results in (C) on the simulated noiseless micrograph.

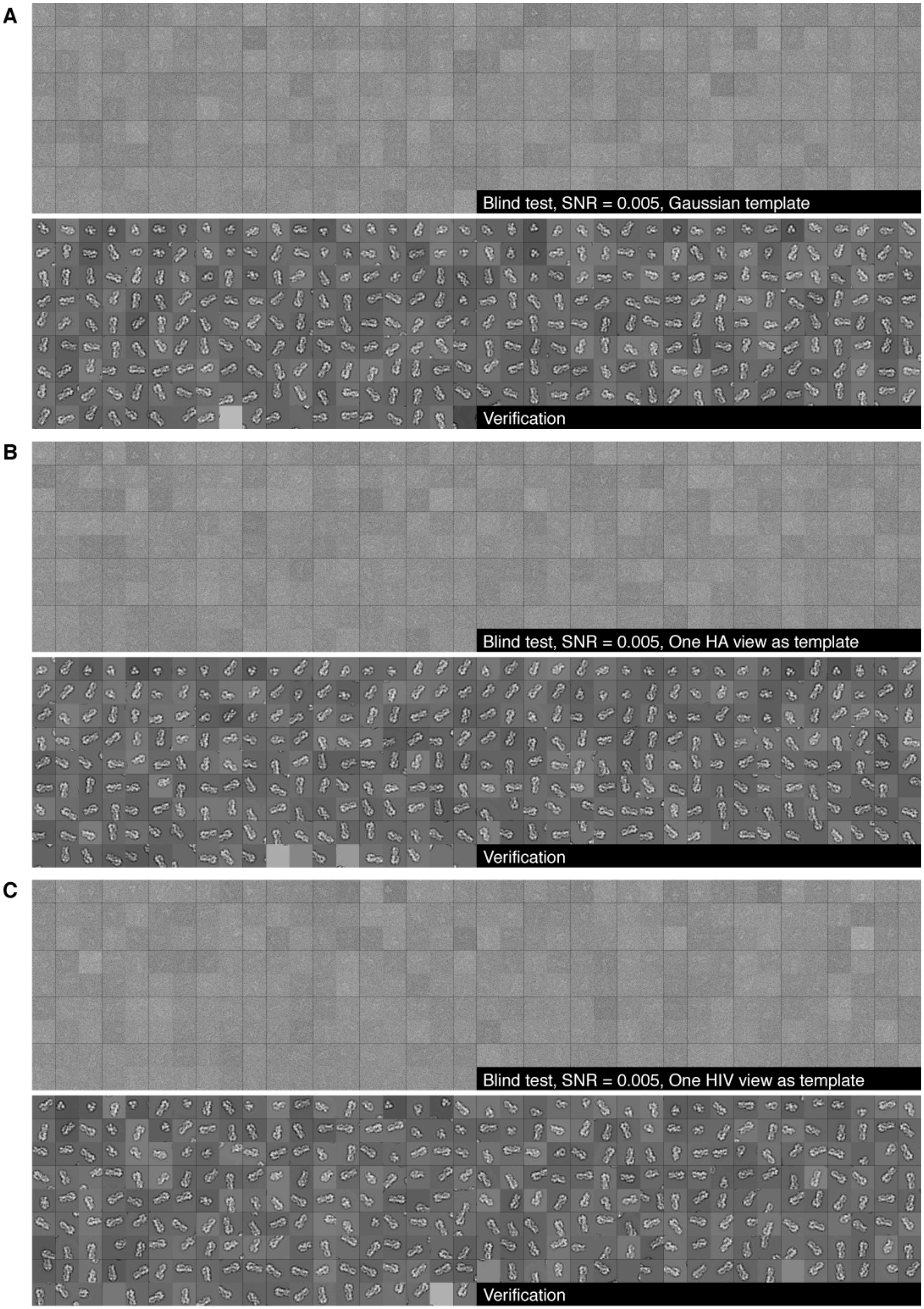
A
Blind test, SNR = 0.005, Gaussian template
Verification
B
Blind test, SNR = 0.005, One HA view as template
Verification
C
Blind test, SNR = 0.005, One HIV view as template
Verification

Figure S4. Comparison of the FLC-based particle-picking results near the critical SNR with different templates. In the upper half of each panel, a gallery of 323 noisy particles boxed out of the influenza virus HA-containing micrographs with an SNR of 0.005 are shown. The lower half of each panel shows a gallery of noiseless particles picked out of the original noiseless micrograph, using the same boxing parameters and in the same sequence as in the corresponding upper panel. This comparison provides a visual verification of the particle-picking performance. The particle-picking templates were a Gaussian circle (A), one projection view of the influenza virus HA trimer (B) and one projection view of the HIV-1 envelope glycoprotein trimer (C).

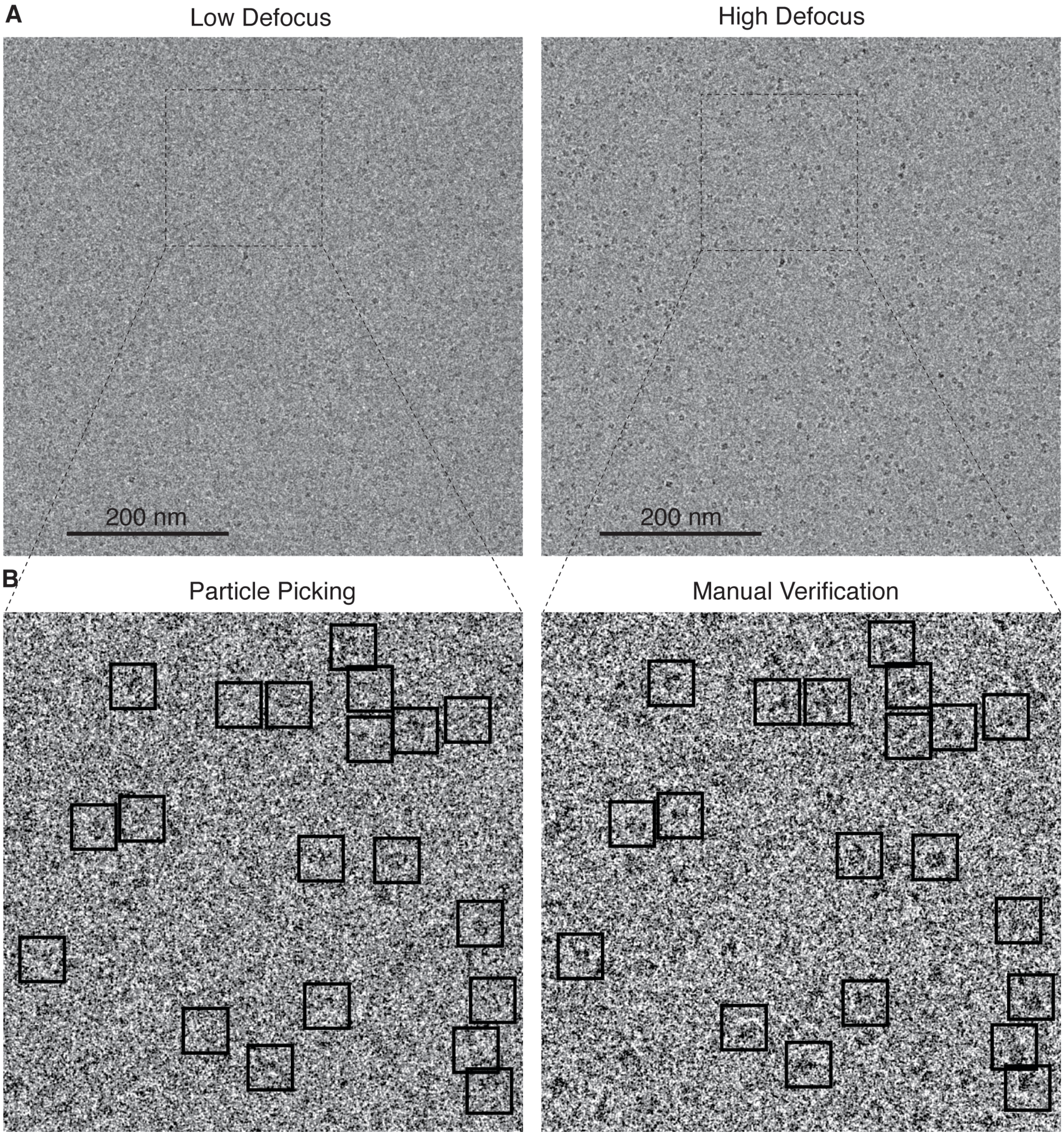

Figure S5. Automated particle picking from low-defocus (close-to-focus) micrographs and manual verification of picked particles from high-defocus (far-from-focus) micrographs. (A) Typical focal pair of micrographs of the 173-kDa glucose isomerase complex taken on a Gatan K2 Summit direct detector camera in the electron counting mode at a defocus of -1.8 μm (left) and -3.3 μm (right). The dimensions of the micrograph are 3696 x 3696 pixels, with a pixel size of 1.74 Å. Each micrograph was exposed to a dose of 10 electrons / $Å^2$. (B) An expanded view of the particle-picking boxes mapped on the low-defocus micrograph on the left, after automated particle picking by the FLC algorithm was applied to

this low-defocus micrograph. In this case, a Gaussian circle was used as the particle-picking template. The same set of boxes is mapped to the higher-defocus micrograph taken in the same sample area for manual verification. The particles picked from the low-defocus micrograph (left) are of low contrast. Nonetheless, the picked particles are mostly true particles, as manually verified by the high-defocus micrograph (right). Note that the particle position moved ~8 nm in the high-defocus micrograph compared to the low-defocus micrograph due to a minor imperfection in the alignment of the rotation center and the camera center. This small movement of the images makes it difficult to directly use the coordinates of the boxed particles from the high-defocus micrograph to pick particles from the low-defocus micrograph without additional alignment of the focal-pair micrographs.

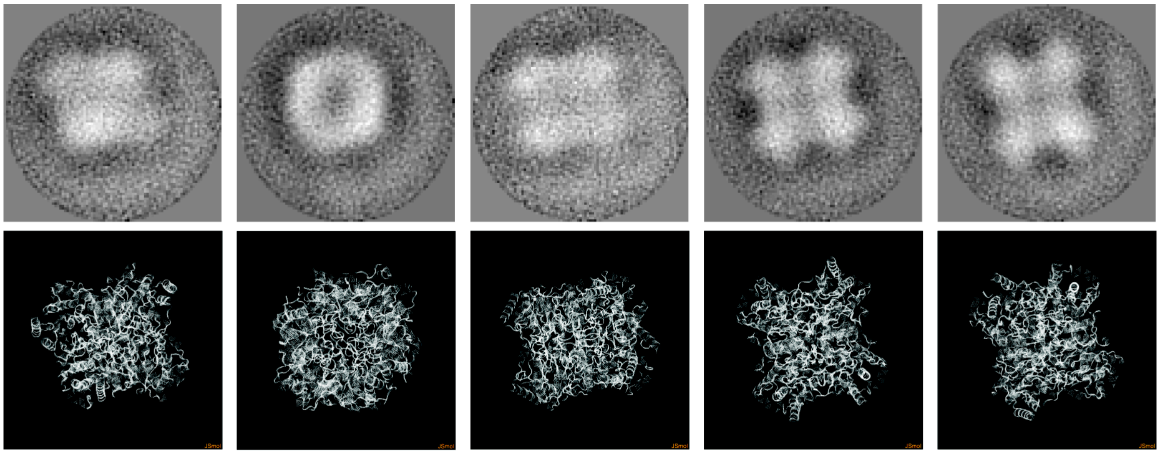

Figure S6. Verification of the class averages after ML classification for the BOF tests on the real cryo-EM data, using the atomic model of the glucose isomerase complex (PDB ID: 1OAD). The class averages shown in the first row resulted from the ML classification for the BOF tests shown in Figure 10B of the main paper. Their corresponding model projections, shown as strand representations of the glucose isomerase atomic model, are presented below each class average. Since all the class averages from the other cases of BOF testing of this data set are quite similar, only one case is shown here for brevity.

Supplementary Protocol

All the computational procedures were implemented in the SPIDER 21.02 (or a higher version) and the XMIPP 2.4 software environment. A detailed step-by-step protocol is described in the following sections.

(1) Simulation of noiseless micrographs and pure noise micrographs

To simulate noiseless micrographs, run the command line:
$ xmipp_phantom_create_micrograph –vol hatrimer.vol –o synmic –density 50 –dim 4098 –N 90 –ctf ctf.ctfparam

Output file:synmic****/synmic****_noiseless.raw

The simulation of a pure noise micrograph is implemented in the SPIDER procedure simul_noise.spi. Run the command line:
$ spider spi/dat @simul_noise

Output file: noisy/noise*****.dat

(2) Add the CTF effect to the noiseless micrographs

This is implemented in the SPIDER procedure add_ctf.spi. Run the command line:
$ spider spi/dat @add_ctf

Input file: synmic****_noiseless.dat (the name extension is changed to dat from raw)
Output file: synmic****_ctf.dat

(3) Add Gaussian noise to the low-contrast micrographs

This is implemented in the SPIDER procedure add_ctf.spi. Run the command line:

$ spider spi/dat @add_noise

Input file: synmic****_ctf.dat
Output file: noisy100/LCM*****.dat

In this process, we generate a different folder for each given SNR. For instance, for an SNR=0.01, we use the folder “noisy100” to store the simulated noisy micrographs. Therefore, for the data set with the CTF effect applied, eight folders were created named: noisy10, noisy20, noisy50, noisy100, noisy200, noisy500, noisy1000, noisy2000, which correspond to SNR = 0.1, 0.05, 0.02, 0.01, 0.005, 0.002, 0.001, 0.0005.

(4) Particle picking with template matching

This was implemented in the standard SPIDER procedure lfc_pick.spi. (This procedure invokes convert_p.spi and pickparticle.spi; these procedures are available from the website http://spider.wadsworth.org/spider_doc/spider/docs/techs/recon/mr.html). Run the command line:
$ spider spi/dat @lfc_pick

Output file: win/winser_*****, win/sel_particle_*****, cords/sndc*****

Generate statics metadata file for the picked particle per micrograph
$ spider spi/dat @pnums_init

Output file: order_picked.dat

Select particles based on the rank of the correlation peaks and a given threshold.
$ spider spi/dat @cutoff

Output file: cutoff/sndc*****.dat

(5) Assemble the particle stack and prepare the data sets for ML alignment

Prepare the data set to be compatible with the input format for XMIPP.
$ spider spi/dat @prep_dataset

Output file: parset/pt*****.dat

(6) Maximum likelihood alignment of the assembled particle data sets

We first generate a metadata file listing all the file names of images to be used for the ML optimization and alignment, by running the command line:
$ xmipp_selfile_create "parset/pt*.dat" > in_images.sel

Next, each image is reduced to a dimension of 60 pixels for a faster calculation in the subsequent ML step, by running the command line:
$ xmipp_scale -i in_images.sel -xdim 60

Each image is normalized for its background noise based on the average and variance of the pixels outside of the circle of 28-pixel diameter, by running the command line:
$ xmipp_normalize -i in_images.sel -background circle 28 -method Ramp

For unsupervised multi-reference ML optimization, run the command line:
$ xmipp_mpi_ml_align2d -i in_images.sel -nref 5 -o ml2d_run01/ml2d -thr 40 -iter 1000 -eps 1e-20 -norm –fast

For unsupervised multi-reference ML optimization with the Gaussian circle as the starting reference, run the command line:
$ xmipp_mpi_ml_align2d -i in_images.sel -ref ref.sel -o ml2d_run02/ml2d -thr 40 -iter 1000 -eps 1e-20 -norm –fast

The ref.sel file contains 5 lines all referring to the same Gaussian circle reference file, as shown below:
$ cat ref.sel

../gaussian_ref.dat 1

../gaussian_ref.dat 1

../gaussian_ref.dat 1

../gaussian_ref.dat 1

../gaussian_ref.dat 1

Supplementary Software

(1) Simul_noise.spi

```
; Simulate purely Gaussian noise micrographs
; ---------- Parameters ----------
[img-sd]           = 1.525E-02  ; standard deviation
[n-s-ratio]        = 50         ; noise-to-signal ratio
[progress-interval] = 10        ; prints progress message to screen every (n)th image
[img-dim]          = 4096       ; micrograph dimension to be simulated
[num-micrographs]   = 200       ; number of pure noise micrographs to be simulated

; ---------- Output files ----------
fr l
[noise_dir]noisy                ; output directory
fr l
[noisy_imgs][noise_dir]/noise*****  ; noisy micrographs

; -------- END BATCH HEADER --------

vm
echo "Simulating pure noise micrographs"; date

vm
echo "if(! -d [noise_dir]) mkdir -p [noise_dir]"|csh

[noisesd]= [img-sd]*sqrt([n-s-ratio])

; loop through the micrographs
do lb1 x10=1,[num-micrographs]
```

```
    if (int(x10/[progress-interval]).eq.x10/[progress-interval]) then
        vm
        echo "Working on image #{*****x11} ({*****x10} out of {*****[num-micrographs]})"
    endif

    ; create noise image
    mo
    [noisy_imgs]x10
    [img-dim],[img-dim]
    R              ; _R_andom distribution
    Y              ; Gaussian-distributed?
    (0,[noisesd])  ; avg, s.d.

lb1
; end particle-loop

ud ice
[particle_list]

vm
echo "Done"; date

en d
```

(2) add_ctf.spi

```
; Simulate Contrast Transfer Function effect in the synthetic noiseless micrographs
; ---------- Parameters ----------
[progress-interval] = 10        ; prints progress message to screen every (n)th image
[img-dim]           = 4096      ; micrograph dimension to be simulated
```

```
[num-micrographs]   = 200        ; number of pure noise micrographs to be simulated
[pad-factor]        = 2          ;

; ---------- Input files ----------
fr l
[parameter_doc]params                  ; parameter doc
fr l
[sel_mic]sel_mic
;fr l
;[ctf_profile]ctfdocs/trapctf***         ; CTF docs
fr l
[noiseless_mic]synmic{****[mic-num]}_noiseless

; ---------- Output files ----------
fr l
[ctf_mic]synmic{****[mic-num]}_ctf0                  ; output directory

; -------- END BATCH HEADER --------

vm
echo "Filtering the micrographs with CTF"; date

; get parameters
ud ic,5,x15   ; pixel size
[parameter_doc]
ud ic,7,x17   ; spherical aberration
[parameter_doc]
ud ic,8,x18   ; source size
[parameter_doc]
ud ic,9,x19   ; defocus spread
[parameter_doc]
```

```
ud ic,12,x22  ; amplitude contrast
[parameter_doc]
ud ic,13,x23  ; Gaussian envelope halfwidth
[parameter_doc]
ud ic,14,x24  ; wavelength
[parameter_doc]
ud ic,15,x25  ; max. spatial frequency
[parameter_doc]
;ud ic,17,x27  ; image dimension
;[parameter_doc]
ud ice        ; close document
[parameter_doc]

; find the number of micrographs
ud n [num-micrographs]
[sel_mic]

[dim-pad] = [img-dim]*[pad-factor]

; loop through the micrographs
do lb1 x10=1,[num-micrographs]

   ud ic x10,[mic-num],[defocus]
   [sel_mic]

   pd
   [noiseless_mic]
   _1        ; OUTPUT
   [dim-pad],[dim-pad],[dim-pad]
   B         ; set background to _B_order
   (1,1,1)   ; top-left coordinates
```

```
; Fourier transform each padded micrograph
ft
_1
_2

; calculate CTF
tf c
_4
x17        ; spherical aberration
[defocus],x24    ; defocus, wavelength
[dim-pad],[dim-pad]    ; x,y-dimensions
x25        ; maximum spatial frequency
(0.005,0)  ; source size, defocus spread
(0,0)      ; astigmatism, azimuth
x22,x23    ; amplitude contrast, Gaussian envelope halfwidth
(-1)       ; sign

; apply CTF to padded micrograph
mu
_2   ; image FT
_4   ; CTF
_5   ; output
*    ; no more images to multiply

; inverse Fourier transform each CTF-corrected FT
ft
_5
_6

; remove padded area in each micrograph
```

```
    wi
    _6   ; input
    [ctf_mic]
    [img-dim],[img-dim],[img-dim]
    (1,1,1)  ; top-left coordinates


lb1
; end particle-loop

ud ice
[sel_mic]

vm
echo "Done"; date

en d
```

(4) add_noise.spi

```
; add Gaussian noise to the simulated noiseless micrographs for a given SNR
; ---------- Parameters ----------
[n-s-ratio]         = 2000         ; noise-to-signal ratio
[progress-interval] = 10         ; prints progress message to screen every (n)th image
[img-dim]           = 4096       ; micrograph dimension to be simulated
[num-micrographs]   = 200        ; number of pure noise micrographs to be simulated

; ---------- Input files ----------
fr l
```

```
[sel_mic]sel_mic
fr l
[noiseless_mic]synmic{****[mic-num]}_ctf0

; ---------- Output files ----------
fr l
[noise_dir]noisyctf2000                    ; output directory
fr l
[noisy_imgs][noise_dir]/LCM*****      ; low-contrast micrographs

; -------- END BATCH HEADER --------

vm
echo "Simulating noisy low-contrast micrographs"; date

vm
echo "if(! -d [noise_dir]) mkdir -p [noise_dir]"|csh



; find the number of micrographs
ud n [num-micrographs]
[sel_mic]

; loop through the micrographs
do lb1 x10=1,[num-micrographs]

   ud ic x10,[mic-num]
   [sel_mic]

   ; find the standard deviation of simulated noiseless micrographs
   fs [max],[min],[img-avg],[img-sd]
```

```
    [noiseless_mic]

    [noisesd]= [img-sd]*sqrt([n-s-ratio])

    if (int(x10/[progress-interval]).eq.x10/[progress-interval]) then
        vm
        echo "Working on micrograph #{*****[mic-num]} ({*****x10} out of {*****[num-
micrographs]})"
    endif

    ; create noise image
    mo
    _1
    [img-dim],[img-dim]
    R             ; _R_andom distribution
    Y             ; Gaussian-distributed?
    (0,[noisesd])  ; avg, s.d.

    ; add to noiseless micrograph
    add
    [noiseless_mic]
    _1
    [noisy_imgs][mic-num]
    *  ; no more files to add


lb1
; end particle-loop

ud ice
[sel_mic]
```

```
vm
echo "Done"; date

en d
```

(5) cutoff.spi

```
; cutoff.spi - automatically select particles according to the preset cutoff value
; convert the coordinates of the selected particle window to the box database file of Boxer in EMAN
; output file lists the particles with peak values above the cutoff value

x98=0.03                    ; The preset cutoff of peak value

x99=323                     ; The maximal number of particles to be selected

; ----------- Input files --------------
FR G
[FILENUMS]../simmgph/sel_mic          ; File numbers

FR G
[picked]order_picked           ; Doc file of all picked particles

FR G
[sndc]coords/sndc{*****x55}     ; Template for doc file with coordinates

; ----------- Output files --------------

FR G
[cutoff]cutoff/sndc{*****x55}  ; Template for doc file with coordinates after cutoff
```

```
FR G
[percent]percent_cutoff        ; Doc file of picked vs selected by cutoff

FR G
[boxer_db]boxer/bxdb{*****x55}  ; Boxer database file to be converted

; ------------- END BATCH HEADER ------------

UD N,x20                    ; Get the number of files
[FILENUMS]

DE
[percent]

SD /     MICROGRAPH    PICKED     SELECTED       %
[percent]

x42=0

DO LB1 x11=1,x20              ; Loop over all micrographs

;   UD x11,x55
;   [FILENUMS]
                            ; x55 is now the micrograph number
  UD x11,x55,x77,x41         ; x77 is the number of picked particles in this micrograph
  [picked]                   ; x41 is the cumulative total of picked particles

  DE
  [cutoff]
```

```
SD /          X          Y       PARTICLE NO.   PEAK HT
[cutoff]

[upper]=x99

IF(x77.LT.x99) THEN
  [upper]=x77
ENDIF

DO LB2 x12=1,[upper]            ; Loop over all picked particles

   UD x12,x78,x79,x80,x81   ; Get the coordinates (x,y), particle no., and peak value
   [sndc]

   IF(x81.GT.x98) THEN       ; Save coordinates of selected particles to doc file
      SD x12,x78,x79,x80,x81
      [cutoff]

      [box_x] = x78 - 90

      [box_y] = x79 - 90

      ; Convert the SPIDER coordinates to a Boxer database file for manual examination
      VM
      echo ' '{****[box_x]} {****[box_y]} 180 180 -3 >> [boxer_db].box

      x42=x42+1             ;Cumulative total
   ENDIF

LB2
```

```
   UD N,x82                    ; x82 is the number of selected particles in this micrograph
   [cutoff]

   x83 = x82/x77               ; Percent of the selected particles

   SD x11,x55,x77,x82,x83
   [percent]

LB1

x43=x42/x41

SD /      TOTALs      PICKED      SELECTED       %
[percent]

x11 = x11 + 1
x55 = 0

SD x11,x55,x41,x42,x43
[percent]

EN D
```

(6) Prep_dataset.spi

```
; Stack selected particles into a file or a directory
; ------------ Input data ---------------------------------------

[max]          = 120;                                  ; Number of micrographs
```

```
[total]          = 1;

; ------------ Input files --------------------------------------

[sel_mic]       = '../simmgph/sel_mic'

[sel_particles]  = 'cutoff/sndc{*****[mic]}'            ; Doc file lists selected particles by micrograph

[win]           = 'win/winser_{*****[mic]}@******'    ; Particle images stacked by micrograph

; --------------- Output files  ------------------------------------

[stk]           = 'parset@*****'                      ; Particle images stacked

[pat]           = 'parset/pt*****'                    ; Particle images in a folder

; -------------- END BATCH HEADER ----------------------------------

UD N,[max]
[sel_mic]

DO [key]=1,[max]                          ; iterate through all micrographs

  UD [key],[mic]                          ; get the micrograph number
  [sel_mic]

  UD N [nums]                             ; get the number of particles selected in each micrograph
  [sel_particles]

  DO [num]=1,[nums]
```

```
      UD [num],[x],[y],[pnum]
      [sel_particles]

      CP
      [win][pnum]
      [stk][total]

      CP
      [win][pnum]
      [pat][total]

      [total] = [total] + 1

   ENDDO

ENDDO

EN D
```
